# Pf Bacteriophage and Their Impact on Pseudomonas Virulence, Mammalian Immunity, and Chronic Infections

**DOI:** 10.3389/fimmu.2020.00244

**Published:** 2020-02-21

**Authors:** Patrick R. Secor, Elizabeth B. Burgener, M. Kinnersley, Laura K. Jennings, Valery Roman-Cruz, Medeea Popescu, Jonas D. Van Belleghem, Naomi Haddock, Conner Copeland, Lia A. Michaels, Christiaan R. de Vries, Qingquan Chen, Julie Pourtois, Travis J. Wheeler, Carlos E. Milla, Paul L. Bollyky

**Affiliations:** ^1^Division of Biological Sciences, University of Montana, Missoula, MT, United States; ^2^Center for Translational Medicine, University of Montana, Missoula, MT, United States; ^3^Center for Biomolecular Structure and Dynamics, University of Montana, Missoula, MT, United States; ^4^Department of Pediatrics, Center for Excellence in Pulmonary Biology, Stanford University, Stanford, CA, United States; ^5^Division of Infectious Diseases and Geographic Medicine, Department of Medicine, Stanford University, Stanford, CA, United States; ^6^Department of Computer Science, University of Montana, Missoula, MT, United States

**Keywords:** bacteriophage, cystic fibrosis, wound, *Pseudomonas aeruginosa*, immunology, infection, Pf phage, Inovirus

## Abstract

Pf bacteriophage are temperate phages that infect the bacterium *Pseudomonas aeruginosa*, a major cause of chronic lung infections in cystic fibrosis (CF) and other settings. Pf and other temperate phages have evolved complex, mutualistic relationships with their bacterial hosts that impact both bacterial phenotypes and chronic infection. We and others have reported that Pf phages are a virulence factor that promote the pathogenesis of *P. aeruginosa* infections in animal models and are associated with worse skin and lung infections in humans. Here we review the biology of Pf phage and what is known about its contributions to pathogenesis and clinical disease. First, we review the structure, genetics, and epidemiology of Pf phage. Next, we address the diverse and surprising ways that Pf phages contribute to *P. aeruginosa* phenotypes including effects on biofilm formation, antibiotic resistance, and motility. Then, we cover data indicating that Pf phages suppress mammalian immunity at sites of bacterial infection. Finally, we discuss recent literature implicating Pf in chronic *P. aeruginosa* infections in CF and other settings. Together, these reports suggest that Pf bacteriophage have direct effects on *P. aeruginosa* infections and that temperate phages are an exciting frontier in microbiology, immunology, and human health.

## Introduction

*Pseud omonas aeruginosa* (*Pa*) is a major human pathogen associated with chronic infections in wounds, burns, and nosocomial settings (1–5). *Pa* is responsible for extensive mortality and billions of dollars in health care costs (6–8). In recognition of this, *Pa* was recently listed as a critical priority pathogen by the World health Organization (WHO) (9).

*Pa* infections are highly problematic in cystic fibrosis (CF), an inherited disease associated with defective ion transport and the accumulation of thick, tenacious airway secretions. Individuals with CF are prone to chronic pulmonary infections that over time lead to poor lung function and increased mortality (10, 11).

*Pa* is particularly pathogenic in CF because of its ability to form robust biofilms (12–18). These are slimy conglomerates of polymers and microbial communities that allow *Pa* to colonize airways and other surfaces (19). *Pa* biofilms are especially tenacious and, once established, very difficult to clear.

Many antibiotics have limited penetration through biofilms (20, 21). Hence, bacteria encased within biofilms are able to tolerate antibiotic concentrations hundreds or thousands of times higher than planktonic bacteria (12, 22–24). Over time, this favors the emergence of antibiotic resistant bacteria (19, 25) and the predominance of multi-drug resistant (MDR) strains (15, 20, 26, 27). Individuals with CF are often infected with *Pa* strains that are resistant to whole classes of anti-pseudomonal antibiotics (28–30), including all oral antibiotic options (15, 31). *Pa* biofilms are also often composed of a heterogeneous bacterial population that includes individuals that are less susceptible to antibiotics, such as metabolically dormant persister cells (32).

*Pa* biofilms also contribute to immune evasion by defying phagocytic engulfment (33–38). Robust biofilm formation by *Pa* contributes to physical impedance of phagocytosis (39, 40) and biofilm polymers also have properties that contribute to immune evasion, including antagonism of complement-mediated opsonization [(35, 37, 41)]. *Pa* also resists efficient bacterial clearance by neutrophil extracellular traps (NETs) (42–44). These are networks of DNA and other polymers released from lysed neutrophils that entrap and destroy bacteria (45, 46). The ability to resist NET-mediated killing (NETosis) is highly strain dependent (42, 43). In the CF airway, ongoing robust immune activation and impaired bacterial clearance perpetuate a vicious cycle of inflammation (47–52).

In light of these effects on antibiotic tolerance and immune evasion, there is great interest in identifying novel biomarkers, virulence factors, and therapeutic targets associated with *Pa* biofilm infections.

Pf bacteriophages, filamentous Inoviruses produced by *Pa* (53, 54), have emerged as a new front in the fight against *Pa* biofilm infections. Here, we review the diverse and surprising ways that Pf phages contribute to the pathogenesis of chronic *Pa* infections in CF and other settings. First, we review the structure, genetics, and epidemiology of Pf phage. Next, we address how Pf phages contribute to *Pa* phenotypes including effects on biofilm formation, antibiotic resistance, and motility. Then, we examine data indicating that Pf modulates mammalian immunity at sites of bacterial infection. Finally, we discuss recent literature implicating Pf in chronic *Pa* infections in CF and other settings. The goal of this work is to provide a comprehensive and integrated resource for those interested in Pf biology and in phage contributions to bacterial pathogenesis.

## *Pa* and Pf Bacteriophages

Pf phages are Inoviruses, a genus of temperate, non-enveloped filamentous viruses. Inoviruses are broadly distributed across all biomes and infect both Gram-positive and Gram-negative bacterial species, and even some Archaea (54). While all lytic phage obligately lyse their bacterial hosts during propagation and most other lysogenic phage typically lyse their bacterial host at some point during their lifecycle, Inoviruses are unique in their ability to establish chronic infection cycles where virions are continuously extruded from the bacterial cell envelope without lysis (55).

Inoviruses like Pf are among the better characterized bacteriophages due to their extensive use in research and industry. Inoviruses are amenable to genetic manipulation and some species, such as M13 (*Escherichia coli*) are widely used in biotechnological applications, such as phage display (56) and as drug carriers (57). Pf phages are also a model system for studying the molecular mechanisms of nucleoprotein assembly and membrane transport (58–68).

In this section, we discuss Pf phage structure, genetics, and lifecycle. We then address the prevalence and diversity of Pf phages amongst *Pa* clinical isolates.

### Pf Virion Structure

**Figure 1:**
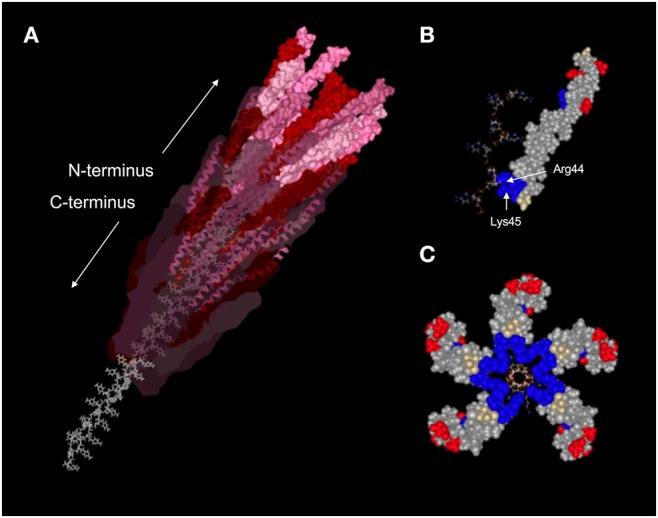
Structure of the Pf1 virion. **(A)** Ribbon diagram with Van der Waals surface representation of a portion of the assembled Pf1 virion (PDBID: 1PFI) showing the helical arrangement of CoaB subunits around the P-form ssDNA viral genome. Subunit surface opacity is decreased along the length of the capsid to highlight the alpha-helical secondary structure of CoaB and the unique conformation of the DNA. Amino acids are colored according to charge, with positively-charged residues in blue, negatively-charged residues in red, and neutral residues in gray. **(B)** Space-filling model of a single CoaB subunit bound to a stretch of cytosines. Arg44 and Lys45 are situated on either side of the DNA backbone and act to stabilize it through electrostatic interactions. **(C)** Cross-sectional view of five CoaB subunits situated around the packaged viral genome. Amino acids are colored by charge as in **(B)**. Image adapted from reference (69).

Pf virions are ~6–7 nm in diameter and vary in length from ~0.8–2 μm, depending on the strain and genome size in question. Like all Inoviruses, Pf virions are composed of thousands of copies of a single major coat protein (p8, CoaB in Pf) with minor coat proteins at either end involved in phage assembly and host recognition (Figure 1) (70–72). Structurally, Inoviruses can be subdivided into two classes based on helical symmetry. Class I Inoviruses are highly symmetrical and include species, such as Fd and M13, which infect *Escherichia coli*. Class II Inoviruses have a twisted, helical symmetry and include Pf phages (73).

Pf phages package a single-stranded circular DNA genome. While most viruses package their genetic material into the smallest conformation possible, Inoviruses like Pf package their genome in the most extended conformation possible. The orientation of the packaged ssDNA Pf phage genome is also unusual; the genome is packaged in a *P* DNA confirmation where the DNA bases are inverted and pointing outwards with the C-terminus of CoaB capsid proteins reaching through the bases to stabilize the backbones of the anti-parallel DNA strands (Figure 1) (74).

Pf virions are relatively stiff filaments with a long persistence length and an overall negative charge density comparable to dsDNA (75). At sufficiently high concentrations, Pf phages, like other filamentous viruses, spontaneously align and assemble liquid crystalline structures (76). The uniform length, diameter, and charge density has made Pf and other Inoviruses a popular model system to study the soft matter physics of colloidal liquid crystals (77) and an effective tool to measure dipolar couplings in NMR structure determinations (78, 79). We discuss how the ability of Pf phages to assemble liquid crystalline structures in biofilms and airway secretions impacts *Pa* infection pathogenesis in a subsequent section below.

### Pf Genome Structure

**Figure 2:**
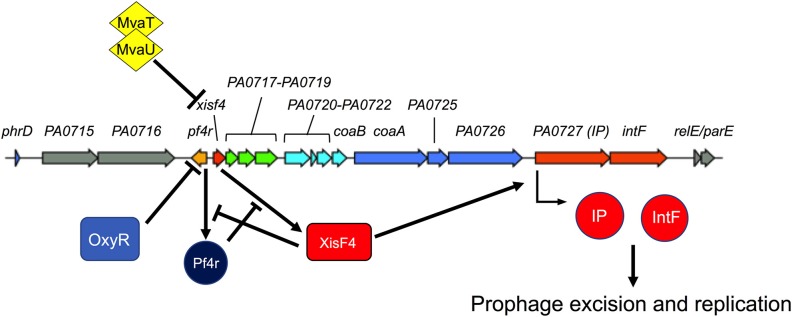
Regulation of Pf replication decisions. The Pf4 prophage from *P. aeruginosa* strain PAO1 is shown. Colored genes correspond to the core Pf genome while gray genes are accessory genes that are variable from strain to strain (see Figure 4 for more details). Pf4r (i.e., the c repressor) maintains lysogeny by repressing the excisionase *xisF4*. XisF4 promotes transcription of an operon encoding both the replication initiation protein PA0727 and integrase IntF. The transcriptional regulator OxyR suppresses *pf4r* while the global histone-nucleosome-like transcriptional regulators MvaT and MvaU suppress *xisF4*. Figure 3 details events in the Pf lifecycle, such as genome replication and assembly of new virions.

Pf phages have a conserved core genome structure encoding genes necessary for DNA replication, virion assembly, and morphogenesis. This is typically present in *Pa* as a prophage integrated into the bacterial chromosome (80). For this review, we refer to Pf gene names and numbers that correspond to the Pf4 prophage integrated into the chromosome of the reference strain *Pa* PAO1 **(**PA0715–PA0729) (Figure 2).

Characterized Pf genes in the core genome include *xisF4* (excisionase), c repressor gene (*pf4r*), *PA0720* (single-stranded DNA binding protein), *PA0723* (*coaB*, major coat protein), *PA0724* (*coaA*, minor coat protein), *PA0726* (morphogenesis protein), *PA0727* (replication initiation protein), and *PA0728* (*intF*, integrase). The functions of these genes are discussed in more detail below. The function of other genes in the Pf core genome remain to be characterized.

Accessory genes flank the core Pf genome (Figure 2) and are known as morons (they add more genes on) (81). While the functions of Pf phage morons are not well-characterized, other phage morons have been shown to reduce virulence factor production and inhibit *Pa* motility (82). Due to the presence of these accessory genes, the overall size of the Pf genome is variable amongst different Pf strains, ranging from ~7–12 kb.

### The Pf Life Cycle

**Figure 3:**
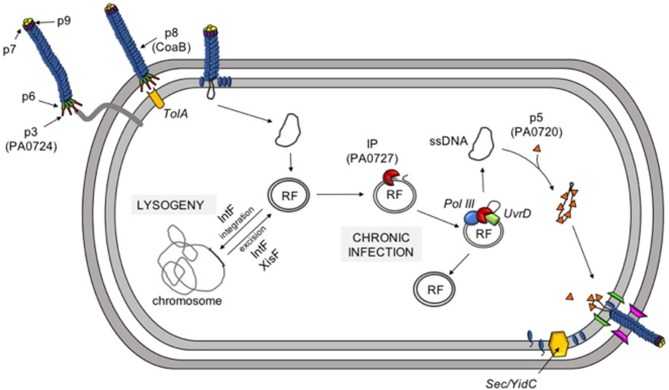
The Pf life cycle. Pf infection is initiated when the minor coat protein p3 (PA0724) binds to type IV pili. Pilus retraction draws the phage into the periplasm where p3 binds the secondary receptor TolA. As the phage moves through the inner membrane, the major coat protein CoaB is removed and deposited in the inner membrane. The ssDNA genome is converted into a dsDNA replicative form (RF) and is either integrated into the host chromosome (lysogeny) or used to initiate a chronic infection. The phage initiator protein (IP, PA0727) and host enzymes (DNA polymerase III and UvrD) create additional copies of RF and the ssDNA infective form (IF) via rolling-circle replication. Newly-produced ssDNA molecules are then coated with protein p5 (PA0720) and targeted to the inner membrane where CoaB, which has been inserted into the inner membrane by the hist Sec/YidC machinery, replaces p5 as the virus is extruded through the cell envelope.

The events around initial infection of bacteria with Inoviruses has been reasonably well-characterized. Pf virions initiate infection by adsorbing to the tip of type IV pili, an extracellular appendage that mediates twitching motility in *Pa* (Figure 3) (83). Adsorption is mediated by the minor coat protein CoaA (PA0724), which is located on the end of the virion. The adsorbed virion is then drawn into the periplasm as the pili retracts where the phage coat protein CoaA contacts the secondary receptor TolA (72). TolA is part of the highly conserved Tol-Pal system that controls membrane integrity and invagination during cell division and is constitutively expressed and readily available for Pf phages to leverage to infect the host cell. CoaB capsid proteins are then drawn off the phage ssDNA genome and retained in the inner membrane while the ssDNA Pf genome is deposited into the cytoplasm. The ssDNA genome is then converted into a circular dsDNA replicative form (RF) by host enzymes and the RF serves as a template for transcription of phage genes.

At this point, the phage must decide whether it will initiate a chronic infection and produce progeny virions or initiate a lysogenic lifecycle and integrate into the bacterial chromosome as a prophage (80). If conditions favor Pf entering a lysogenic lifecycle after infecting a naïve bacterial host, the RF can be integrated into the bacterial chromosome as a prophage by the Pf-encoded site-specific tyrosine recombinase IntF (84).

Recent work has shed light on the molecular details for how Pf phages maintain lysogeny or initiate a chronic infection cycle. Like other phages, Pf phages respond to host stress. For example, Pf5 replication is strongly induced when the substrate binding protein DppA1 (involved in peptide utilization) is inactivated (85). Although the mechanism is not known, it is thought that Pf5 senses nutrient limitation of its host through DppA1 and in response, initiates the chronic infection cycle. Given the importance of Pf phages for Pa biofilm formation and pathogenesis discussed below, the further characterization of factors that drive production of Pf phages is likely to be an area of active research.

Pf phages, like several other phages, also respond to oxidative stress. When *Pa* is exposed to H_2_O_2_, cysteine residues in the OxyR transcriptional regulator are oxidized (86). Oxidized OxyR then binds to the Pf4 prophage between *PA0716* and *PA0719* (86). The binding of OxyR to this site likely suppresses the c repressor gene *pf4r*, which maintains lysogeny and confers immunity to superinfection by exogenous Pf virions. Pf4r promotes lysogeny and superinfection exclusion by repressing transcription of the Pf excisionase gene *xisF4* (84) (Figure 2).

When *pf4r* is suppressed, as it is by OxyR, or when sufficiently high titers of infecting Pf virions overwhelm Pf4r, as may happen during superinfection, XisF4 is free to promote the transcription of the operon encoding the replication initiator protein (PA0727) and IntF (84). The initiator protein binds to RF DNA at the origin of replication, recruits the host helicase UvrD and DNA polymerase III, and initiates rolling circle replication (87). Rolling circle replication not only produces new copies of the dsDNA RF, but also new ssDNA Pf genomes. The newly synthesized ssDNA genomes are quickly coated by numerous copies of the single stranded-binding protein PA0720, stabilizing and protecting the ssDNA species until it can be packaged into a new virion.

While the RF and ssDNA pools expand in the bacterial cytoplasm, structural Pf proteins, such as CoaB are produced. CoaB is translated with an N-terminal leader peptide that targets the CoaB preprotein to the inner membrane. After CoaB is inserted in the inner membrane in the correct orientation, it is processed into its mature form by host Sec/YidC enzymes (88). CoaB monomers interact with membrane-associated morphogenesis machinery (which likely includes the morphogenesis protein PA0726) to package the ssDNA Pf genome. As CoaB is incorporated into the growing virion, PA0720 is displaced and the nascent phage particle is extruded through the cell envelope (Figure 3).

### Superinfection of *Pa* by Pf Phages

Once extruded from the host cell, Pf virions likely encounter a host that is already lysogenized by a Pf prophage. This is especially true when bacterial densities are high, as they are in biofilms. Superinfection occurs when exogenous Pf virions successfully infect a bacterial cell already lysogenized by Pf. Even though Pf (and other Inoviruses) can replicate without lysing their bacterial host, Pf superinfection can result in bacterial lysis. At present it is unclear how Pf superinfection causes bacterial lysis. It is possible that during superinfection more Pf virions are produced than an individual host cell can handle. For example, CoaB monomers or other Pf proteins could accumulate in the inner membrane to sufficiently high levels such that the cytoplasmic membrane is destabilized.

Superinfection by Pf phages plays important roles in *Pa* biofilm development. Evaluation of the within-population genetic diversity of *Pa* PAO1 during biofilm formation revealed that the Pf4 population diversified at an evolutionary rate comparable to RNA viruses, far faster than the rest of the genome (89). In conjunction with the diversification of the Pf4 population, titers of superinfective Pf4 virions as high as 10^11^ plaque forming units per milliliter of biofilm effluent were observed. Interestingly, all mutations in Pf4 were within or upstream of the c repressor gene *pf4r*, suggesting that superinfective Pf phages lose the ability to self-regulate their replication. Lastly, the study by McElroy *et al*. also found that mutations in type IV pili genes also accumulated in *Pa* biofilm populations, most likely in response to selective pressures exerted by the expansion of the Pf phage population (89).

*Pa* can suppress Pf phage through the histone-like nucleoid-structuring (H-NS) proteins MvaT and MvaU (83). MvaT and MvaU are global regulators of gene expression and coordinately suppress AT-rich elements in the *Pa* genome (90). The *Pa* genome is G-C-rich (~66% GC content) and MvaT and MvaU offer a mechanism for *Pa* to incorporate xenogenic DNA (such as phages and other horizontally acquired genetic material with a relatively high AT-content) into its genome in a regulated way. Because MvaT and MvaU regulate the same target genes, *Pa* can tolerate the loss of either *mvaT* or *mvaU*; however, inactivation of both *mvaT* and *mvaU* is lethal to *Pa* (90). A transposon mutagenesis study discovered that the conditional depletion of MvaT in a Δ*mvaU* background induced Pf4 replication that resulted in the death of *Pa* (83). That study also demonstrated that Pf4 uses type IV pili as a cell surface receptor to initiate infection. Interestingly, when type IV pili were inactivated by deleting the *pilY* gene, depletion of MvaT in a Δ*mvaU* background was tolerated, even though the Pf4 prophage was induced and infectious Pf4 virions were produced (83). This observation indicates that superinfection was required to cause cell death.

These observations are consistent with a model where within *Pa* biofilms, populations of Pf phages with inactivated c repressor genes expand and Pf virions are produced in abundance. These virions could then superinfect *Pa* at a sufficiently high multiplicity of infection to overwhelm the superinfection exclusion mechanism mediated by Pf4r proteins produced by the resident Pf prophage. Under these conditions, individuals with type IV pili mutations would be selected for as they would be resistant to Pf superinfection.

### Pf Diversity and Prevalence Amongst *Pa* Isolates

**Figure 4:**
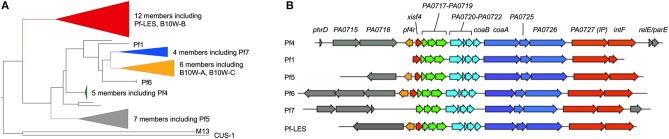
Phylogenic analysis of Pf prophages. **(A)** Forty representative full-length Pf prophage instances were manually collected from the Pseudomonas Genome Database (91). Two DNA sequences encoding Protein III (PA0724) from Inovirus strains M13 and CUS-1 (*E. coli*) were added to serve as an outgroup for phylogenetic rooting. A multiple sequence alignment of these viral sequences was produced using MAFFT (93) (v7.215, default parameters), then manually trimmed to include only the well-aligned region containing Pf phage core proteins. A maximum-likelihood estimate of the strain phylogeny was produced with FastTree (94) (v2.1.3, default parameters), and visualized with FigTree (v1.4.4). **(B)** Representative full-length Pf prophage sequences were manually collected from the Pseudomonas Genome Database. Strain Pf4 was used as a reference; open reading frames identified in other Pf strains were color-coded based on genome location relative to Pf4. Gray genes indicate variable moron sequences (see text) outside of the core Pf genome.

Routine whole genome sequencing of *Pa* isolates has revealed that Pf prophages are prevalent amongst *Pa* isolates. In one study, out of 241 *Pa* strains, ~60% were lysogenized by Pf (80). Of 2,226 genomes available through the Pseudomonas Genome Database (91), 52% encoded a Pf prophage (92). A phylogenetic analysis of Pf prophage sequences revealed that most known Pf prophages encode their own integrase *intF* (Figure 4). However, Pf1, the first Pf strain isolated in 1966 (95), does not encode an intact integrase and replicates episomally (it cannot enter a lysogenic lifecycle). Strains of Pf that do not integrate into the bacterial chromosome would not be detected by whole genome sequencing. Therefore, the prevalence of Pf phages amongst *Pa* isolates is likely underestimated.

Many Pf prophages integrate into bacterial tRNA genes, as is true with other temperate phages (96). For example, the Pf4 prophage infecting *Pa* strain PAO1 integrates into the tRNA-Gly gene *PA0729.1*. It is worth noting that the subline strain MPAO1 harbors both Pf4 and a second similar Pf prophage designated Pf6 integrated into the tRNA-Met gene *PA4673.1* (97). Another example is Pf7, which is integrated into the tRNA-Met gene PSPA7_5361 in *Pa* strain PA7. Some Pf strains, however, integrate into other genomic locations. Strain Pf5, which infects *Pa* strain PA14, integrates between the C32 tRNA thiolase (*PA14_48870*) and *dnaJ* (*PA14_49040*). Other Pf prophages integrate into a homologous site in other strains of *Pa* including Pf-LES, which infects *Pa* epidemic strain LES58 (80) and an unclassified Pf strain infecting *Pa* strain 19660 (91).

We have observed that several strains of *Pa* are infected by multiple Pf prophages. This is in contrast to many species of phages (e.g., lambda) where, due to superinfection exclusion mechanisms, a preexisting phage prevents secondary infections by the same or closely related phage. As noted above, MPAO1 harbors both Pf4 and Pf6. Other strains, such as *Pa* B10W harbors three Pf prophages. Two of the three Pf prophages integrated into the B10W chromosome are genetically distinct from each other (Figure 4) indicating that the Pf prophages of BW10 were obtained from distinct infections by different strains of Pf phages along with potential duplications of the same Pf prophage. Given the roles Pf phages play in biofilm formation and infection pathogenesis (see below), it would be interesting to compare the fitness of *Pa* strains lysogenized by a single Pf prophage compared to strains lysogenized with several Pf phages.

## Pf Effects on *Pa* Phenotypes and Fitness

Infection of the CF airway with *Pa* starts with planktonic (free living), variants often originating from the environment. Over time, as bacteria adapt to the CF airway, they environment, they form a biofilm (98). In this section we address the contribution of Pf phages to *Pa* biofilm formation and to other phenotypes involved in *Pa* pathogenicity.

### Pf Phages and Small Colony Variant (SCV) Morphology

In *Pa* PAO1 biofilms, bacterial cells shed from the biofilm display a small colony variant (SCV) morphology after they are grown on solid agar (99–101). The production of SCVs is correlated with the emergence of superinfective Pf4 phage. SCVs revert back to a “fuzzy” wild-type colony morphology after subculture in Pf phage-free lysogeny broth (101), indicating that the SCV morphology is a phenotypic response to Pf superinfection and not due to heritable mutations. Because Pf superinfection both suppresses type IV pili-mediated twitching motility and induces a severe growth lag in *Pa*, it is possible that the SCV morphology associated with chronic Pf infection is a result of reduced growth coupled to inhibited twitching motility. The emergence of SCVs from *Pa* biofilms appears to be variable amongst *Pa* strains—*Pa* PA14 biofilms do not shed cells that form SCVs even though Pf5 virions were present at high densities in PA14 biofilms (102). The underlying mechanism for this discrepancy is unknown, but it may relate to the different integration sites of Pf4 and Pf5 and/or the different moron genes encoded by Pf4 and Pf5 (Figure 4B).

### Pf Phages and *Pa* Biofilm Formation

Upregulation of Pf phage genes are a common feature of *Pa* biofilms, as found in a metanalysis of transcriptional data from *Pa* biofilms grown under various laboratory settings (103). In addition to gene expression data, *Pa* biofilms grown under different conditions (dripflow, chemostat, colony biofilms, etc.), are all associated with the production of abundant superinfective Pf phages at titers as high as 10^11^ PFU/ml (89, 99, 100, 104). Furthermore, semiviscous (105) and anaerobic conditions (106) that mimic environments in biofilms and in late-stage CF-airways were found to induce Pf phages, although the mechanisms behind these induction cues are unknown. Collectively, these observations indicate that Pf phages are consistently induced in *Pa* biofilms, raising the possibility that Pf phages play important roles in biofilm formation. Indeed, Pf phages contribute to *Pa* biofilms in diverse and surprising ways.

**Figure 5:**
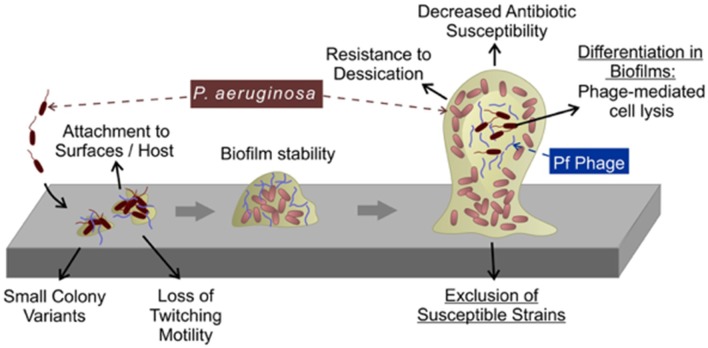
Pf phages contribute to biofilm formation. During the early stages of biofilm formation, Pf phages promote bacterial adhesion to surfaces. During later stages, Pf phages serve as structural components in the biofilm matrix where they promote tolerance to desiccation and cationic antimicrobials. Superinfection by Pf phages (see text) promotes differentiation of the biofilm by inducing bacterial lysis in a subpopulation of cells deep within the biofilm.

The formation of a biofilm is initiated by bacterial attachment to a surface followed by the formation of microcolonies (107). As microcolonies differentiate and grow, voids form in the middle of these microcolonies that are created by cell lysis (100). Cell lysis is accompanied by the release of DNA into the extracellular space (eDNA), which adds structural integrity to the biofilm (108). The presence of superinfective Pf phages in the biofilm effluent correlates with cell lysis and eDNA release prior to the dispersion phase of the biofilm lifecycle (109, 110) (Figure 5). To this point, when Pf4 prophage was deleted from the *Pa* PAO1 chromosome, bacterial lysis and eDNA release were not observed (99). Bacterial lysis and eDNA release caused by superinfective Pf phages appears to be a regulated process; the two-component regulator BfmR was reported to suppress Pf phage-mediated cell lysis through PhdA (PA0691), an antitoxin homolog in the Phd (prevent-host-death) family of proteins (111). Deleting *bfmR* caused the premature induction of superinfective Pf phages, increased bacterial lysis and eDNA release, phenotypes opposite to the ΔPf4 prophage mutant. It is possible that PhdA induces Pf replication by interacting with the ParE plasmid stabilization system encoded by Pf4 (Figure 4B) (110). However, experimental evidence for this interaction is lacking.

The presence of Pf phages is also associated with phenotypes linked to the pathogenicity of *Pa* biofilms (Figure 5). Expectorated sputum from CF patients infected by *Pa* contain ~10^6^-10^9^ Pf virions per ml (92, 101). Filamentous Pf phages can dramatically increase the viscosity of CF airway polymers, such as mucin and DNA (92, 101). Furthermore, Pf phage-producing biofilms are more adherent to surfaces compared to *Pa* not producing Pf virions (92, 99–101). Thus, Pf virions could benefit *Pa* infecting CF airways by reducing bacterial clearance from the lung. Consistent with this idea, *Pa* superinfected by Pf established a non-invasive infection phenotype in a mouse model of pneumonia and did not disseminate from the lung whereas *Pa* not producing Pf virions disseminated to distal tissues, suggesting that Pf phages contribute to the establishment of conditions that promote localized chronic infection (112). Other filamentous phages, such as MDAΦ that infects *Neisseria meningitidis* likewise promotes adhesion to epithelial cells promoting host colonization (113). Thus, filamentous phages may play key roles in host colonization and infection initiation in various bacterial infections.

Rice *et al*. demonstrated that biofilms formed by the Pf phage-deficient mutant ΔPf4 were more sensitive to disruption by the detergent SDS relative to the parental strain *Pa* PAO1 lysogenized by Pf4 (99), suggesting that filamentous Pf phages contribute to the structural integrity of biofilms. As discussed above, Pf phages are stiff, negatively charged filaments that spontaneously assemble liquid crystalline structures. Our previous work revealed that Pf virions accumulate in the biofilm matrix where they spontaneously align and give the biofilm matrix liquid crystalline properties (101, 114). Bacteria living in a liquid crystalline biofilm matrix were resistant to desiccation, possibly because the highly ordered liquid crystalline matrix was better able to retain water compared to non-crystalline matrices. Furthermore, the negative charge carried by Pf phages facilitates the binding and sequestration of cationic aminoglycoside antibiotics and cationic antimicrobial peptides (92, 101). This binding capacity was enhanced when Pf phages were in the liquid crystalline phase, allowing *Pa* to tolerate otherwise lethal doses of cationic antibiotics (101, 114). In clinical settings, tolerance to antimicrobials could promote the emergence of individuals with fixed antibiotic resistance, thus limiting treatment options and promoting chronic infection. Indeed, filamentous bacteriophages are associated with chronic *Pa* lung infections and antibiotic resistance in CF (92). Because all Inoviruses are composed of acidic coat proteins, filamentous bacteriophages may be an overlooked structural component of the biofilm matrix in other bacterial species.

Other ways that Pf phages could benefit *Pa* biofilms is by excluding susceptible strains (104). Consistent with this, recent work demonstrates that *Pa* isolates collected from older CF patients tend to be lysogenized by Pf compared to isolates collected earlier in disease (92). This observation raises the possibility that *Pa* strains not harboring Pf prophages may be replaced by strains lysogenized by Pf as the disease progresses to later stages. Future studies investigating the role of Pf phages in the establishment and maintenance of *Pa* lineages in chronic CF airway infections may provide insight into how *Pa* dominates CF airway infections.

## Pf Effects on Mammalian Immunity

There is increasing interest in the impact of phages on human immunology. This trend parallels the resurgence of efforts to develop phage therapies to treat chronic/multidrug-resistant bacterial infections (115, 116) as well as the rapid expansion of research into endogenous phages residing on and within the human host (117–119). Much of this work has focused on CF and the viral pathogens of *Pa* (120, 121).

In this section, we discuss what is known about Pf and its impact on mammalian immunity. First, we discuss Pf and its impact on phagocytosis and bacterial clearance. Next, we address Pf effects on cytokine production and the inflammatory milieu. Finally, we discuss how Pf biology relates to what is known about mammalian immunity and other endogenous and exogenous phages and highlight the outstanding questions around these interactions.

### Pf Effects on Bacterial Clearance by Phagocytes

The ability of *Pa* to establish residency and persist in the human lung is dependent upon the bacterium's efficacy at evading innate immune attacks, particularly neutrophil- and alveolar macrophage-mediated anti-bactericidal defenses (122–126). *Pa* must also contend with multiple pattern-recognition receptors (PRRs) including Toll-like receptors 4 (TLR4), 5 (TLR5), and 9 (TLR9) that recognize bacterial pathogen-associated molecular patterns (PAMP) molecules including lipopolysaccharide (LPS) (127, 128), flagellin (128, 129), and DNA containing CpG motifs (130), respectively. PAMP-mediated stimulation of phagocytosis is the primary host defense against *Pa* infection, as evidenced by the fact that both animal models and people with defects in phagocytic cell function are highly susceptible to *Pa* infection (125, 131, 132).

*Pa* possess a diverse set of virulence factors that impede phagocytosis by both neutrophils and macrophages (38, 133). Many of these factors impact the machinery of phagocytosis, including inflammatory cytokine production, cellular recruitment, and bacterial engulfment (38, 134, 135). These effects on phagocytic clearance contribute to *Pa* immune evasion and persistence within infected tissues (136).

We recently reported that Pf directly inhibits phagocytosis and bacterial clearance (137). Pf causes a significant decrease in the number of *Pa* phagocytosed by murine-derived dendritic cells (DC), macrophages, and human U937-derived and primary macrophages (137). Additionally, murine phagocytes exposed to Pf4 phagocytosed fewer dead *E. coli* particles compared to saline controls, indicating that the inhibitory effects of Pf on phagocytosis are not limited to engulfment of *Pa* (137).

Pf phage also influence bacterial phenotypes in ways that are likely to prevent efficient phagocytosis. As discussed above, some strains of Pf phage contribute to the emergence of SCV strains (99, 110) which are known to drive exopolysaccharide-dependent resistance to macrophage phagocytosis (138). Furthermore, Pf contributions to biofilm formation, adhesiveness, and increased polymer viscosity could be expected to interfere with phagocytosis given that biofilms formed by *Pa* and other organisms are known to prevent opsonization and engulfment of the bacteria within (38, 139–141). Finally, the same structural attributes that promote liquid crystal assembly by Pf phages also drives bacterial aggregation (142) and loss of motility (112), features associated with diminished phagocytosis (135). We discuss reduced phagocytic uptake of bacteria through the lens of CF in later sections.

### Pf Effects on Cytokine Production and the Inflammatory Milieu

Inflammatory cytokines are critical to host immune clearance of *Pa*. It is well-established that TNF in particular plays a crucial role in stimulating phagocytosis (143, 144) and in polarizing macrophages toward an M1 phenotype associated with efficient bacterial clearance.

We recently reported that Pf phages are associated with decreased cellular production of TNF (137). These effects were associated with enhanced type 1 interferon levels. Consistent with a critical role for TNF in bacterial clearance, we observed that supplemental TNF could correct Pf effects on phagocytosis and bacterial clearance *in vivo* (137). Pf also suppressed the production of other cytokines and chemokines important for bacterial clearance, including CXCL1 and IL-17. Conversely, Pf increased production of IL-12 and type 1 interferon (112, 137). Fd phage, an *Inovirus* that infects *E. coli*, did not affect TNFa production or phagocytosis (137). The differences in the immunomodulatory properties of Pf and fd phages is unclear, but may relate to differences in coat protein/virion structure or amino acid composition.

Pf-dependent modulation of cytokine production by immune cells was associated with intracellular uptake of Pf phage. We reported that Pf is taken up by human and mouse macrophages, B cells, and dendritic cells within endosomal vesicles. These results are consistent with reports that other phages are internalized by mammalian cells, though those reports mostly implicated phagocytosis rather than endocytosis (145, 146). Further, there are indications that large numbers of phages are directionally transcytosed across epithelial layers into system circulation (147), raising the possibility that phages regularly modulate immune responses and impact human health and disease.

We observed that intracellular Pf phage reduce TNF production by triggering the viral pattern recognition receptor TLR3. In bone-marrow derived macrophages (BMDMs) from TLR2^−/−^, TLR9^−/−^, and MyD88^−/−^ mice, Pf phage reduced TNF upon LPS stimulation, but in TRIF^−/−^, TLR3^−/−^, and IFNAR^−/−^ BMDMs, Pf phage had no significant effect on TNF production (137).

**Figure 6:**
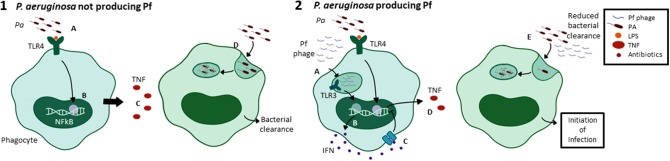
Pf phages trigger viral pathogen recognition pathways leading to reduced phagocytosis. Phagocytosis is the major way that *Pa* infections are cleared by the immune system. In the absence of Pf phages, *Pa*-derived LPS is recognized by TLR4 on the surface of alveolar macrophages (**1**A), triggering NFkB activation and pro-inflammatory cytokine production (**1**B). TNF, a major pro-inflammatory effector, is secreted as a result (**1**C). This drives *Pa* phagocytosis by alveolar macrophages, leading to bacterial clearance (**1**D). In the presence of a *Pa* strain that produces Pf phages, phage particles are internalized in endosomes (**2**A), triggering TLR3 activation and production of interferons (**2**B). IFNα/β binds IFNAR on the cell surface (**2**C) and negatively regulates pro-inflammatory cytokine production, decreasing TNF secretion (**2**D). This results in decreased phagocytic engulfment of *Pa* (**2**E). Together, this supports a model where intracellular Pf phage triggers viral pattern recognition pathways that antagonize bacterial clearance.

Pf also impacts the polarization of macrophages in the inflammatory milieu. *Pa* PAO1 superinfected with Pf4 caused decreased expression of genes associated with pro-inflammatory M1 macrophage polarization, including Nos2, IL-12, and IFN-g; along with increased expression of cytokines associated with anti-inflammatory M2 macrophage polarization, including IL-10, Nos1, and Arg1 (112, 148). Together with the aforementioned effects on cytokine production, these data indicate that internalization of Pf phages triggers anti-viral pattern recognition receptors that antagonize antibacterial immunity (Figure 6).

### The Emerging Picture of Phage/Immune Interactions

While our review here has focused on Pf phage, there is a growing body of literature on phage/immune interactions, primarily in the context of phage therapy. Much of that work is outside of the scope of this review and there are several recent, excellent summaries of that research (149–151). Nonetheless, it is worth addressing the commonalities and differences of this larger body of work in relation to Pf phages.

The finding that Pf suppresses phagocytosis contrasts with reports that lytic phages promote phagocytosis and interact synergistically with phagocytes (152–156). There are also suggestions that some species of phage may promote the opsonization of bacteria by adhering to the bacterial surface and acting as a target for antibody binding (157). In addition, there are reports that phages may be induced when their bacterial host is phagocytosed, lysing the bacteria and promoting clearance (149, 158). Together, these data suggest that lytic phages may promote opposite effects on bacterial clearance compared to Pf. The mechanistic basis for the differences and similarities seen between lytic and lysogenic phages is unclear, particularly since lytic phage are also internalized by eukaryotic cells (159). However, there are recent examples; enteric Caudovirales phages (tailed phages that package linear dsDNA) initially purified for therapeutic use were found to stimulate increased production of pro-inflammatory cytokines (119). In the context of inflammatory bowel diseases, these phages directly exacerbated disease. While further, direct comparisons are needed, these distinctions could be important for the future prospects of lytic phage therapy.

While the impact on bacterial clearance may differ, the impact of cytokine production and the inflammatory milieu appears to be more similar. Studies with lytic phage have generally reported similar, anti-inflammatory effects *in vitro* (160–162) as well as *in vivo* (163, 164). In particular, lytic phage may also dampen the inflammatory effects of LPS and other bacterial products (165, 166). This stands in contrast to some data implicating other, endogenous eukaryotic viruses in chronic inflammation (167) and, it has been theorized, in protection against pathogenic viral infections (168).

## The Impact of Pf Phage on *Pa* Infections in CF and Other Settings

Recent reports have implicated Pf phage in clinical outcomes associated with *Pa* infections. Here, we discuss this literature and the potential role of Pf phage in CF lung infections. First, we discuss the links between Pf and clinical outcomes in CF. Next, we discuss the development of vaccines that target Pf to prevent infections with *Pa* and the implications of our work on lytic phage therapy. Finally, we address the relevance of our work on filamentous phage to studies on the lung microbiome.

### Pf Phage in CF Airway Infections

CF is a genetic disease where the cystic fibrosis transmembrane conductance regulator (CFTR) gene is mutated. In healthy individuals, the CFTR protein is expressed highly on the apical surface of epithelial cells. In people with CF, CFTR is absent or dysfunctional leading to an imbalance of chloride secretion and sodium absorption which causes dehydration of the airway surface (169–171). Without well-hydrated airway surface liquid, impaired ciliary beating and mucous transport lead to mucus stasis and buildup of viscous mucous in the airways. This in turn sets off a vicious cycle of bacterial infection and neutrophil dominated inflammation, resulting in severe and progressive obstructive pulmonary disease with significant morbidity and mortality (172, 173).

Lung disease in CF begins early in life with recurrent and chronic bacterial infections (172, 174). As the disease progresses, the airways of most patients with CF become colonized by *Pa* (175). In its early stages, *Pa* is intermittently recovered in respiratory cultures, before progressing to a chronic state (176, 177). By adulthood, nearly 60% of CF patients have chronic *Pa* infections (178, 179). Over time, *Pa* becomes a driving force in clinical outcomes in CF lung infections. Chronic endobronchial infection with *Pa* is associated with poor lung function, increased frequency of symptomatic episodes of pulmonary exacerbation, and increased mortality (180–183). While *Pa* eradication antibiotic protocols are now standard of care, success is unfortunately variable and not sustained (184, 185).

We recently identified an association between chronic *Pa* infections in CF patients and Pf phage. In 474 samples collected from a cohort of 34 Danish CF patients infected with Pa, we found that 44.5% were positive for Pf phage (92). In a cross-sectional study of 58 *Pa* positive CF patients followed at the Stanford CF Clinic, 36% were Pf phage positive. In this cohort, detection of Pf phage was associated with older age, advanced lung disease, worse disease exacerbations, and antibiotic resistance (92). As additional evidence for the damaging effects of these altered responses, we also observed a correlation between the presence of Pf in sputum and the activity of the neutrophil enzyme Elastase (186), a known marker of airway destruction and bronchiectasis in CF. Further, we found that immune dysfunction correlates of the presence of Pf with sputum cytokine profiling revealing higher levels of IL12p70 and lower levels of ENA78, RESISTIN and TRAIL (186). These changes suggest disrupted immune function in response to infection (187–192). Broadly speaking, these data are consistent with the enhanced virulence associated with Pf production in a mouse model of *Pa* lung infection and with reports by ourselves and others describing contributions of Pf to chronic infection phenotypes (99, 101, 110, 112).

**Figure 7:**
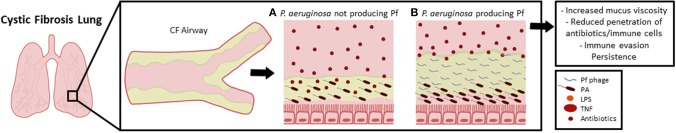
Pf phages contribute to the pathogenesis of *Pa* airway infections in CF. The thick mucus coating the airways in a CF lung facilitates colonization with *Pa* and other bacterial pathogens. Even in the absence of Pf phages, *Pa* forms tenacious biofilms which protect bacterial cells from phagosomal clearance and resist penetration by antibiotics **(A)**. However, Pf phages when present organize the polymer-rich biofilm into crystalline, higher order structures **(B)**, This drives sputum viscosity, adhesiveness, and resistance to desiccation. It also enhances the antibiotic tolerance and immune evasion of bacterial colonies within biofilms, thereby contributing to increased bacterial burden. In this way Pf phages enhance the pathogenesis of *Pa* airway infections in CF.

The effects of Pf on clinical outcomes were also associated with an increased rate of genetically-encoded antibiotic resistance to several anti-pseudomonal antibiotics—particularly aztreonam, amikacin, and meropenem. These same antibiotics were also sequestered by Pf phage *in vitro* whereas ciprofloxacin was not (92) (Figure 7). This raises the intriguing possibility that over time, treatment with these antibiotics favors the acquisition of Pf(+) strains of *Pa*. Consistent with this, we observed that only 1 of 11 pediatric patients in our cohort was Pf(+), whereas 20 of 46 adult patients were (92). Further, 10 of 10 CF patients who underwent lung transplantation were Pf(+) (101). In light of these data, we have proposed that *Pa* strains that produce Pf phage may have a selective advantage and be disproportionately represented in the lungs of older patients with CF. These data also suggest that it may be possible to use the presence of Pf phage to inform antibiotic choices.

These findings in *Pa*-infected CF patients echo the results of our recent investigations into the role of Pf phages in *Pa* chronic wound infections. In a prospective cohort study of 113 patients with wound infections we found that *Pa* was detected in 37 patients and that 25 of these (68%) were positive for Pf phage. Pf(+) strains of *Pa* were more likely than Pf(−) strains to be present in chronic wounds (137).

Together, these data suggest that Pf phages may contribute to clinical outcomes in *Pa* infections in CF as well as in other clinical contexts.

### The Development of Therapies That Target Pf Phages to Prevent *Pa* Infections

An effective vaccine against *Pa* would represent an important component in our armamentarium against this deadly bacterium. To date, there are no FDA approved vaccines for the prevention of *Pa* infections, though several are in various stages of development (193). Most of the vaccine candidates that have been evaluated have been against major components of *Pa* itself, including LPS, exopolysaccharides, extracellular and outer membrane proteins, flagella, or pili (193). Given the prevalence of Pf amongst *Pa* clinical isolates and its contribution to immune evasion and biofilm formation (92, 137), Pf represents an attractive and novel target for immunization against *Pa*.

The ability of antibodies to neutralize bacteriophages has been described and phage neutralizing antibodies have been detected in the sera of several species (151, 194, 195). Studies of sera from patients receiving phage therapy against *Staphylococcus aureus* showed the production of antibodies against the phages in question (196). In a different study, sera collected from healthy individuals contained neutralizing antibodies against naturally occurring bacteriophage (151, 197). On the other hand, other studies did not find evidence of phage targeted antibodies in phage therapy recipients (198). It may be that some phage are more antigenic than others in certain hosts, though the lack of standardized methodologies for these studies may also be a factor underlying these heterogenous results.

Our recent work demonstrated that immunization against Pf provided protection against *Pa* infections in mice. Immunization was performed using a peptide derived from the CoaB coat protein of Pf phage conjugated to the carrier protein keyhole limpet hemocyanin (KLH). Mice vaccinated with this novel formulation were able to clear *Pa* infection challenges more often than unvaccinated mice. Similar rates of clearance were obtained in the setting of passive immunization with monoclonal antibodies developed against the CoaB peptide. Further, anti-CoaB monoclonal antibodies (mAB) were effective in promoting opsonization of *Pa* (137). These data suggest that Pf phage is a potential candidate for a preventative vaccine against *Pa*.

Much work remains to be done before such vaccines could become a realistic possibility. In particular, the effectiveness of this strategy against clinical isolates, it's specificity for *Pa*, and a detailed mechanism of action in regards to how it promotes bacterial clearance will all be necessary. Nonetheless, the prospect of such a vaccine is enticing. It is tempting to consider that this could be administered to individuals diagnosed with CF before they develop chronic *Pa* infections. Similarly, individuals with diabetes could be immunized before they develop their first *Pa* wound infection.

### The Relevance of Pf Studies for Lytic Phage Therapy

With the rising prevalence of antibiotic-resistant bacteria, novel approaches to deal with bacterial infections are an urgent need (199). Phage therapy is the application of bacteria-specific viruses to combat uncontrolled and undesired bacteria, such as those associated with infectious disease (200). Phage therapy for *Pa* infections has more than 50 years of history but there is a recent resurgence of interest in this approach (201–203). There are several potential advantages of phage therapy that make it an appealing alternative or adjunct to conventional antibiotics. These include the lytic activity of the phages, auto dosing, low inherent toxicity, minimal disruption of normal flora, narrower potential for inducing resistance, lack of cross-resistance with antibiotics, rapid discovery, formulation and application versatility, and effects on biofilm clearance (204). There nonetheless remain many potential issues that need to be addressed, including demonstrations of safety and efficacy in double-blinded, case controlled clinical trials.

One important potential consideration is the nature of the immunological response induced by phages. As briefly summarized above, there are some suggestions that phage-mediated effects on the inflammatory milieu are generally well-tolerated without major side effects [(119, 137, 151, 155, 161, 205)]. However, this remains to be examined in a rigorous and systematic manner, ideally in the context of parallel evaluations of safety and efficacy. A better understanding of the interactions of phage—host—human interactions will provide valuable insight into predicting the outcome of phage therapy (151).

Data on filamentous, lysogenic phage are of unclear relevance for phage therapy studies. As noted above, diverse phage seem to be have broadly non-inflammatory effects. However, to our knowledge lytic phage have not been reported to be directly immune suppressive in the way that Pf phages are reported to be. Indeed, there are reasons to think that the effects of lytic vs. temperate phage on local immunity may prove to be quite distinct. Not only are the viruses in question different genetically and phenotypically, but lytic phage are likely to be encountered in the context of bacterial detritus from lysed cells whereas Pf phage may not. It is also unclear to what extent different phage are taken up intracellularly or trigger viral PRRs. Clearly the comparative effects of different phage is an important question for future research. In the meantime, in the absence of evidence, we favor a cautious approach to extrapolating results from work on Pf phages to other phages.

### Pf Phages in the Context of the Larger Lung Phageome

The virome is increasingly recognized as an integral part of the human microbiome, interacting with other members of the microbial community to shape host phenotypes (206). Bacteriophages make up a majority of this virome (207), but their impact on the larger microbial community within the lung and on lung health remain understudied. New sequencing methods have made metagenomics a more affordable and reliable approach to doing so (208) but substantial technical barriers remain (209, 210).

The lung phageome may be distinct in different disease states. One study suggested that in healthy individuals, the composition of the sputum phageome is highly heterogenous and may reflect organisms in inhaled air or oral flora. In contrast, the sputum phageome of in CF individuals was highly similar (211, 212). Given recent findings regarding microbial heterogeneity within different lung regions of CF patients (213), it may be important to revisit the question of the lung phageome with more in depth sampling regimens. Nonetheless, this common footprint is remarkable. It may be that selective bacterial growth conditions lead to common, relatively stable microbial community compositions over time (212, 214) with metabolic activities that reflect host characteristics (211, 214, 215). Supporting this idea, Willner et al. found that the viral community in the lungs of a CF patient's non-CF spouse shared taxonomy with the CF virome but had the metabolic profile of a non-CF virome (211).

The contributions of Pf phages to the overall lung microbiome are undefined but potentially substantial. The impact of Pf phages on biofilm formation, antibiotic sequestration, virulence factors, and modulation of immunity described above are likely to influence not only *Pa* phenotypes but also the overall ecology of the lung.

Pf phages can also affect the flow of resources within the biofilm. We reported that Pf phages impact the growth of fungal organisms, specifically Aspergillus and Candida. As noted above, Pf phages bind to free iron through charge-based interactions, much in the same way that they sequester antibiotics, and this deprives fungi of this critical resource (216, 217). We also reported that Pf phages change the material properties of biofilms, making them far more viscous (101). In addition to increasing the tenacity and thickness of CF sputum, this is also likely to impact the movement and distribution of microbes within the biofilm. It seems probable that we are only scratching the surface of the ways in which Pf phages may influence microbial ecology.

Future studies of the lung microbiome in Pf(+) and Pf(−) subjects are likely to be highly informative. The integrated, simultaneous study of both bacterial and phage communities, including prophages and their induction (218) will be important.

## Concluding Remarks and Areas for Future Investigation

Several themes emerge from this review. The relationship between Pf phages and *Pa* appears to be highly evolved and is clearly an important factor in *Pa* fitness. Despite the substantial energetic cost of producing so many phage particles, Pf phages are nonetheless common among *Pa* strains in multiple contexts, including in the CF lung.

Pf phage also contribute to *Pa* pathogenesis in multiple models, tissues, and human disease contexts. In the context of CF lung infections, Pf phages contribute to the impaired bacterial clearance and heightened inflammation that are characteristic of the disease. It seems likely that Pf contributions to antibiotic tolerance are a major driver in this setting. However, Pf effects on biofilm formation, antibiotic tolerance, microbial ecology, and immune modulation may be important as well.

It may be possible to exploit this biology for therapeutic gain. The anti-Pf vaccine discussed here is the clearest example of this. However, other applications are also possible. For example, the presence of Pf phages may also have potential to inform antibiotic treatment regimens. Manipulating the relationships between bacterial pathogens and their phages may also be an exciting therapeutic frontier.

Much about Pf phage biology remains unknown. While Pf phages are relatively well-characterized compared to most other phages, much remains unknown about their biology. In particular, the function of many Pf phage genes, the factors that govern Pf production, and the impact of Pf phages on *Pa* virulence factors are all promising areas of study that are likely to play important roles in *Pa* pathogenesis.

While the molecular details of the Pf lifecycle are reasonably well-defined, many questions remain regarding how the Pf lifecycle intersects with the functioning of their bacterial host. One obvious gap in our knowledge is the function of the uncharacterized genes in the core Pf genome. Another understudied aspect of Pf biology is their ability to bind not only cationic antimicrobials, but also multivalent cations like iron (75, 216, 217). Iron is a limiting nutrient at infection sites and the ability of Pf phages to bind and sequester this essential nutrient may have important implications in infection pathogenesis. Lastly, the ability of these filamentous phages to assemble liquid crystals in the extracellular environment likely has implications for the host-pathogen interactions.

The evolutionary origins of phage/mammalian host interactions are unclear. Recent work revealed that the (cGAS)-STING pathway, an innate antiviral defense mechanism in animals, has evolutionary roots with bacterial anti-phage defense mechanisms (219). Thus, phages may be triggering ancient highly conserved immune responses in animal cells. It seems unlikely that Pf phage evolved to manipulate mammalian immunity, given that mammals are not a natural host for *Pa*. Instead, it may be that these effects of Pf on animal immune systems enable *Pa* to likewise avoid predation by phagocytic predators, such as amoeba in the environment. Similar effects on amoeba predation have been hypothesized for other *Pa* factors that impact mammalian phagocytosis, including exotoxins, alginate secretion, and the regulation of motility (136).

The relationship between Pf and *Pa* may also be dynamic and in flux. Bacteria-phage relationships can evolve from parasitic interactions to mutualistic relationships over time with the most extreme examples giving rise to new bacterial functions. Examples include the type VI secretion system (220) and pyocins (221) of *Pa*. The Pf prophage may likewise eventually evolve from an independent virus into a bacterial operon encoding a filamentous structural element of the extracellular biofilm matrix that also has immunomodulatory properties.

Many outstanding questions remain to be addressed in regards to the impact of phage on mammalian immunity. Inoviruses like Pf are widespread amongst bacteria (54) and it is unclear whether the immune effects outlined here for Pf are present in association with other filamentous phage. There are some hints that this may be the case. M13 filamentous phages produced by *E. coli* can be internalized *in vivo* (222) and *in vitro* (223). As mentioned above, a filamentous phage (MDAφ) produced by *N. meningitidis* may likewise increase host-cell colonization, bacterial aggregation, and virulence (113). Given the extensive heterogeneity between and even within Inoviruses (54), it seems likely that here again further investigation will reveal substantial differences.

Relatively little is known about adaptive immune responses to Pf and other phages. Most studies suggest that humoral immunity (antibodies) against phage exists and is rapidly induced upon exposure to phage (194, 196, 197, 224, 225); the impact of these on phage therapy is an active area of investigation and may be particularly important for phage therapy (226).

In summary, Pf phages are an exciting frontier in microbiology, immunology, and human health. Further investigations are likely to yield additional insights into both *Pa* pathogenesis as well as novel therapeutic and prophylactic treatments.

## Author Contributions

PS, EB, MK, LJ, VR-C, MP, JV, NH, CC, LM, CV, QC, JP, TW, CM, and PB all contributed to the writing, graphics, and editing of this work.

### Conflict of Interest

The authors declare that the research was conducted in the absence of any commercial or financial relationships that could be construed as a potential conflict of interest.

## References

[1] Casemore DP. Foodborne protozoal infection. Lancet. (1990) 336:1427–32. 10.1016/0140-6736(90)93115-6 1978883

[2] Lipsky BA Berendt AR Deery HG Embil JM Joseph WS Karchmer AW. Diagnosis and treatment of diabetic foot infections. Clin Infect Dis. (2004) 39:885–910. 10.1086/424846 15472838

[3] Tredget EE Shankowsky HA Rennie R Burrell RE Logsetty S. Pseudomonas infections in the thermally injured patient. Burns. (2004) 30:3–26. 10.1016/j.burns.2003.08.007 14693082

[4] Gardner SE Frantz RA. Wound bioburden and infection-related complications in diabetic foot ulcers. Biol Res Nurs. (2008) 10:44–53. 10.1177/1099800408319056 18647759 PMC3777233

[5] Jarbrink K Ni G Sonnergren H Schmidtchen A Pang C Bajpai R. Prevalence and incidence of chronic wounds and related complications: a protocol for a systematic review. Syst Rev. (2016) 5:152. 10.1186/s13643-016-0329-y 27609108 PMC5017042

[6] Morales E Cots F Sala M Comas M Belvis F Riu M. Hospital costs of nosocomial multi-drug resistant *Pseudomonas aeruginosa* acquisition. BMC Health Serv Res. (2012) 12:122. 10.1186/1472-6963-12-122 22621745 PMC3412693

[7] Sansgiry SS Joish VN Boklage S Goyal RK Chopra P Sethi S. Economic burden of *Pseudomonas aeruginosa* infection in patients with cystic fibrosis. J Med Econ. (2012) 15:219–24. 10.3111/13696998.2011.638954 22084956

[8] Blanchette CM Noone JM Stone G Zacherle E Patel RP Howden R. Healthcare cost and utilization before and after diagnosis of *Pseudomonas aeruginosa* among patients with non-cystic fibrosis bronchiectasis in the U.S. Med Sci. (2017) 5:20. 10.3390/medsci5040020 29099036 PMC5753649

[9] Tacconelli E Carrara E Savoldi A Harbarth S Mendelson M Monnet DL. Discovery, research, and development of new antibiotics: the WHO priority list of antibiotic-resistant bacteria and tuberculosis. Lancet Infect Dis. (2018) 18:318–27. 10.1016/S1473-3099(17)30753-3 29276051

[10] Folkesson A Jelsbak L Yang L Johansen HK Ciofu O Hoiby N. Adaptation of *Pseudomonas aeruginosa* to the cystic fibrosis airway: an evolutionary perspective. Nat Rev Microbiol. (2012) 10:841–51. 10.1038/nrmicro2907 23147702

[11] Malhotra S Hayes D Jr Wozniak DJ. Cystic Fibrosis and *Pseudomonas aeruginosa*: the Host-Microbe Interface. Clin Microbiol Rev. (2019) 32:e00138-18. 10.1128/CMR.00138-18 31142499 PMC6589863

[12] Costerton JW Stewart PS Greenberg EP. Bacterial biofilms: a common cause of persistent infections. Science. (1999) 284:1318–22. 10.1126/science.284.5418.1318 10334980

[13] Jesaitis AJ Franklin MJ Berglund D Sasaki M Lord CI Bleazard JB. Compromised host defense on *Pseudomonas aeruginosa* biofilms: characterization of neutrophil and biofilm interactions. J Immunol. (2003) 171:4329–39. 10.4049/jimmunol.171.8.4329 14530358

[14] Moreau-Marquis S Stanton BA O'Toole GA. *Pseudomonas aeruginosa* biofilm formation in the cystic fibrosis airway. Pulm Pharmacol Ther. (2008) 21:595–9. 10.1016/j.pupt.2007.12.001 18234534 PMC2542406

[15] Hirsch EB Tam VH. Impact of multidrug-resistant *Pseudomonas aeruginosa* infection on patient outcomes. Expert Rev Pharmacoecon Outcomes Res. (2010) 10:441–51. 10.1586/erp.10.49 20715920 PMC3071543

[16] Hoiby N Ciofu O Bjarnsholt T. *Pseudomonas aeruginosa* biofilms in cystic fibrosis. Future Microbiol. (2010) 5:1663–74. 10.2217/fmb.10.125 21133688

[17] Zhao G Hochwalt PC Usui ML Underwood RA Singh PK James GA. Delayed wound healing in diabetic (db/db) mice with *Pseudomonas aeruginosa* biofilm challenge: a model for the study of chronic wounds. Wound Repair Regen. (2010) 18:467–77. 10.1111/j.1524-475X.2010.00608.x 20731798 PMC2939909

[18] Percival SL Suleman L Vuotto C Donelli G. Healthcare-associated infections, medical devices and biofilms: risk, tolerance and control. J Med Microbiol. (2015) 64(Pt 4):323–34. 10.1099/jmm.0.000032 25670813

[19] Ishida H Ishida Y Kurosaka Y Otani T Sato K Kobayashi H. *In vitro* and *in vivo* activities of levofloxacin against biofilm-producing *Pseudomonas aeruginosa*. Antimicrob Agents Chemother. (1998) 42:1641–5. 10.1128/AAC.42.7.1641 9660997 PMC105659

[20] Hoiby N Bjarnsholt T Givskov M Molin S Ciofu O. Antibiotic resistance of bacterial biofilms. Int J Antimicrob Agents. (2010) 35:322–32. 10.1016/j.ijantimicag.2009.12.011 20149602

[21] Bjarnsholt T The role of bacterial biofilms in chronic infections. APMIS Suppl. (2013). (136):1–51. 10.1111/apm.12099 23635385

[22] Ciofu O Bagge N Hoiby N. Antibodies against beta-lactamase can improve ceftazidime treatment of lung infection with beta-lactam-resistant *Pseudomonas aeruginosa* in a rat model of chronic lung infection. APMIS. (2002) 110:881–91. 10.1034/j.1600-0463.2002.1101207.x 12645667

[23] Parsek MR Singh PK. Bacterial biofilms: an emerging link to disease pathogenesis. Annu Rev Microbiol. (2003) 57:677–701. 10.1146/annurev.micro.57.030502.090720 14527295

[24] Chiang WC Nilsson M Jensen PO Hoiby N Nielsen TE Givskov M. Extracellular DNA shields against aminoglycosides in *Pseudomonas aeruginosa* biofilms. Antimicrob Agents Chemother. (2013) 57:2352–61. 10.1128/AAC.00001-13 23478967 PMC3632962

[25] Meers P Neville M Malinin V Scotto AW Sardaryan G Kurumunda R. Biofilm penetration, triggered release and *in vivo* activity of inhaled liposomal amikacin in chronic *Pseudomonas aeruginosa* lung infections. J Antimicrob Chemother. (2008) 61:859–68. 10.1093/jac/dkn059 18305202

[26] Drenkard E Ausubel FM. Pseudomonas biofilm formation and antibiotic resistance are linked to phenotypic variation. Nature. (2002) 416:740–3. 10.1038/416740a 11961556

[27] Levin-Reisman I Ronin I Gefen O Braniss I Shoresh N Balaban NQ. Antibiotic tolerance facilitates the evolution of resistance. Science. (2017) 355:826–30. 10.1126/science.aaj2191 28183996

[28] Obritsch MD Fish DN MacLaren R Jung R. Nosocomial infections due to multidrug-resistant *Pseudomonas aeruginosa*: epidemiology and treatment options. Pharmacotherapy. (2005) 25:1353–64. 10.1592/phco.2005.25.10.1353 16185180

[29] Lister PD Wolter DJ Hanson ND. Antibacterial-resistant *Pseudomonas aeruginosa*: clinical impact and complex regulation of chromosomally encoded resistance mechanisms. Clin Microbiol Rev. (2009) 22:582–610. 10.1128/CMR.00040-09 19822890 PMC2772362

[30] Chmiel JF Aksamit TR Chotirmall SH Dasenbrook EC Elborn JS LiPuma JJ. Antibiotic management of lung infections in cystic fibrosis. I The microbiome, methicillin-resistant *Staphylococcus aureus*, gram-negative bacteria, and multiple infections. Ann Am Thorac Soc. (2014) 11:1120–9. 10.1513/AnnalsATS.201402-050AS 25102221 PMC5467101

[31] Bassetti M Vena A Croxatto A Righi E Guery B. How to manage *Pseudomonas aeruginosa* infections. Drugs Context. (2018) 7:212527. 10.7573/dic.212527 29872449 PMC5978525

[32] Grassi L Di Luca M Maisetta G Rinaldi AC Esin S Trampuz A. Generation of persister cells of *Pseudomonas aeruginosa* and *Staphylococcus aureus* by chemical treatment and evaluation of their susceptibility to membrane-targeting agents. Front Microbiol. (2017) 8:1917. 10.3389/fmicb.2017.01917 29046671 PMC5632672

[33] Buret A Cripps AW. The immunoevasive activities of *Pseudomonas aeruginosa*. Relevance for cystic fibrosis. Am Rev Respir Dis. (1993) 148:793–805. 10.1164/ajrccm/148.3.793 8368651

[34] Leid JG Willson CJ Shirtliff ME Hassett DJ Parsek MR Jeffers AK. The exopolysaccharide alginate protects *Pseudomonas aeruginosa* biofilm bacteria from IFN-gamma-mediated macrophage killing. J Immunol. (2005) 175:7512–8. 10.4049/jimmunol.175.11.7512 16301659

[35] Morris MR Doull IJ Dewitt S Hallett MB. Reduced iC3b-mediated phagocytotic capacity of pulmonary neutrophils in cystic fibrosis. Clin Exp Immunol. (2005) 142:68–75. 10.1111/j.1365-2249.2005.02893.x 16178858 PMC1809487

[36] Anderson GG O'Toole GA. Innate and induced resistance mechanisms of bacterial biofilms. Curr Top Microbiol Immunol. (2008) 322:85–105. 10.1007/978-3-540-75418-3_5 18453273

[37] Mishra M Byrd MS Sergeant S Azad AK Parsek MR McPhail L. *Pseudomonas aeruginosa* Psl polysaccharide reduces neutrophil phagocytosis and the oxidative response by limiting complement-mediated opsonization. Cell Microbiol. (2012) 14:95–106. 10.1111/j.1462-5822.2011.01704.x 21951860 PMC4466118

[38] Alhede M Bjarnsholt T Givskov M Alhede M. *Pseudomonas aeruginosa* biofilms: mechanisms of immune evasion. Adv Appl Microbiol. (2014) 86:1–40. 10.1016/B978-0-12-800262-9.00001-9 24377853

[39] Vishwanath S Ramphal R Guay CM DesJardins D Pier GB. Respiratory-mucin inhibition of the opsonophagocytic killing of *Pseudomonas aeruginosa*. Infect Immun. (1988) 56:2218–22. 10.1128/IAI.56.9.2218-2222.1988 3137161 PMC259552

[40] Hansch GM Brenner-Weiss G Prior B Wagner C Obst U. The extracellular polymer substance of *Pseudomonas aeruginosa*: too slippery for neutrophils to migrate on? Int J Artif Organs. (2008) 31:796–803. 10.1177/039139880803100907 18924091

[41] Boucher JC Yu H Mudd MH Deretic V. Mucoid *Pseudomonas aeruginosa* in cystic fibrosis: characterization of muc mutations in clinical isolates and analysis of clearance in a mouse model of respiratory infection. Infect Immun. (1997) 65:3838–46. 10.1128/IAI.65.9.3838-3846.1997 9284161 PMC175548

[42] Young RL Malcolm KC Kret JE Caceres SM Poch KR Nichols DP. Neutrophil extracellular trap (NET)-mediated killing of *Pseudomonas aeruginosa*: evidence of acquired resistance within the CF airway, independent of CFTR. PLoS ONE. (2011) 6:e23637. 10.1371/journal.pone.0023637 21909403 PMC3164657

[43] Shan Q Dwyer M Rahman S Gadjeva M. Distinct susceptibilities of corneal *Pseudomonas aeruginosa* clinical isolates to neutrophil extracellular trap-mediated immunity. Infect Immun. (2014) 82:4135–43. 10.1128/IAI.02169-14 25047845 PMC4187885

[44] Yoo DG Winn M Pang L Moskowitz SM Malech HL Leto TL. Release of cystic fibrosis airway inflammatory markers from *Pseudomonas aeruginosa*-stimulated human neutrophils involves NADPH oxidase-dependent extracellular DNA trap formation. J Immunol. (2014) 192:4728–38. 10.4049/jimmunol.1301589 24740504 PMC4032287

[45] Brinkmann V Zychlinsky A. Neutrophil extracellular traps: is immunity the second function of chromatin? J Cell Biol. (2012) 198:773–83. 10.1083/jcb.201203170 22945932 PMC3432757

[46] Baums CG von Kockritz-Blickwede M. Novel role of DNA in neutrophil extracellular traps. Trends Microbiol. (2015) 23:330–1. 10.1016/j.tim.2015.04.003 25913613

[47] Bonfield TL Panuska JR Konstan MW Hilliard KA Hilliard JB Ghnaim H. Inflammatory cytokines in cystic fibrosis lungs. Am J Respir Crit Care Med. (1995) 152(6 Pt 1):2111–8. 10.1164/ajrccm.152.6.8520783 8520783

[48] Hartl D Latzin P Hordijk P Marcos V Rudolph C Woischnik M. Cleavage of CXCR1 on neutrophils disables bacterial killing in cystic fibrosis lung disease. Nat Med. (2007) 13:1423–30. 10.1038/nm1690 18059279

[49] Mayer-Hamblett N Aitken ML Accurso FJ Kronmal RA Konstan MW Burns JL. Association between pulmonary function and sputum biomarkers in cystic fibrosis. Am J Respir Crit Care Med. (2007) 175:822–8. 10.1164/rccm.200609-1354OC 17234902 PMC2720115

[50] Elizur A Cannon CL Ferkol TW. Airway inflammation in cystic fibrosis. Chest. (2008) 133:489–95. 10.1378/chest.07-1631 18252915

[51] Ulrich M Worlitzsch D Viglio S Siegmann N Iadarola P Shute JK. Alveolar inflammation in cystic fibrosis. J Cyst Fibros. (2010) 9:217–27. 10.1016/j.jcf.2010.03.001 20347403 PMC2883667

[52] Sagel SD Wagner BD Anthony MM Emmett P Zemanick ET. Sputum biomarkers of inflammation and lung function decline in children with cystic fibrosis. Am J Respir Crit Care Med. (2012) 186:857–65. 10.1164/rccm.201203-0507OC 22904182 PMC3530222

[53] Salmond GP Fineran PC. A century of the phage: past, present and future. Nat Rev Microbiol. (2015) 13:777–86. 10.1038/nrmicro3564 26548913

[54] Roux S Krupovic M Daly RA Borges AL Nayfach S Schulz F. Cryptic inoviruses revealed as pervasive in bacteria and archaea across Earth's biomes. Nat Microbiol. (2019) 4:1895–906. 10.1038/s41564-019-0510-x 31332386 PMC6813254

[55] Rakonjac J Bennett NJ Spagnuolo J Gagic D Russel M. Filamentous bacteriophage: biology, phage display and nanotechnology applications. Curr Issues Mol Biol. (2011) 13:51–76. 21502666

[56] Mimmi S Maisano D Quinto I Iaccino E. Phage display: an overview in context to drug discovery. Trends Pharmacol Sci. (2019) 40:87–91. 10.1016/j.tips.2018.12.005 30606501

[57] Ju Z Sun W. Drug delivery vectors based on filamentous bacteriophages and phage-mimetic nanoparticles. Drug Deliv. (2017) 24:1898–908. 10.1080/10717544.2017.1410259 29191048 PMC8241185

[58] Raoult D Perrin G Hechemy KE Vestris G San-Kale M. [Meningitis from Lyme disease, diagnosed in Marseilles]. Presse Med. (1985) 14:1615. 2931714

[59] Colovic R Jankovic R Popovic M Krivokapic Z. [Desmoid tumors]. Srp Arh Celok Lek. (1987) 115:605–8. 2978633

[60] Rao S Coleman PS. Control of DNA replication and cell growth by inhibiting the export of mitochondrially derived citrate. Exp Cell Res. (1989) 180:341–52. 10.1016/0014-4827(89)90062-1 2492469

[61] Nambudripad R Stark W Opella SJ Makowski L. Membrane-mediated assembly of filamentous bacteriophage Pf1 coat protein. Science. (1991) 252:1305–8. 10.1126/science.1925543 1925543

[62] Welsh LC Symmons MF Marvin DA. The molecular structure and structural transition of the alpha-helical capsid in filamentous bacteriophage Pf1. Acta Crystallogr D Biol Crystallogr. (2000) 56(Pt 2):137–50. 10.1107/S0907444999015334 10666593

[63] Tsuboi M Kubo Y Ikeda T Overman SA Osman O Thomas GJ Jr. Protein and DNA residue orientations in the filamentous virus Pf1 determined by polarized Raman and polarized FTIR spectroscopy. Biochemistry. (2003) 42:940–50. 10.1021/bi020566v 12549913

[64] Thiriot DS Nevzorov AA Zagyanskiy L Wu CH Opella SJ. Structure of the coat protein in Pf1 bacteriophage determined by solid-state NMR spectroscopy. J Mol Biol. (2004) 341:869–79. 10.1016/j.jmb.2004.06.038 15288792

[65] Goldbourt A Gross BJ Day LA McDermott AE. Filamentous phage studied by magic-angle spinning NMR: resonance assignment and secondary structure of the coat protein in Pf1. J Am Chem Soc. (2007) 129:2338–44. 10.1021/ja066928u 17279748

[66] Park SH Marassi FM Black D Opella SJ. Structure and dynamics of the membrane-bound form of Pf1 coat protein: implications of structural rearrangement for virus assembly. Biophys J. (2010) 99:1465–74. 10.1016/j.bpj.2010.06.009 20816058 PMC2931714

[67] Straus SK Scott WR Schwieters CD Marvin DA. Consensus structure of Pf1 filamentous bacteriophage from X-ray fibre diffraction and solid-state NMR. Eur Biophys J. (2011) 40:221–34. 10.1007/s00249-010-0640-9 21082179 PMC5545983

[68] Clinical toxicology II Clinical toxicology II. Strategies for clinical laboratory management: horizontal and vertical integration in hospital laboratories. Clin Lab Med. (1990) 10:441–641. 2253442

[69] Lorieau JL Day LA McDermott AE. Conformational dynamics of an intact virus: order parameters for the coat protein of Pf1 bacteriophage. Proc Natl Acad Sci USA. (2008) 105:10366–71. 10.1073/pnas.0800405105 18653759 PMC2492469

[70] Virus Taxonomy Ninth report of the international committee on taxonomy of viruses. In: King AMQ Adams MJ Carstens EB Lefkowitz EJ editors. Virus Taxonomy. San Diego, CA: Elsevier (2012). p. 1281–325.

[71] Marvin DA Symmons MF Straus SK. Structure and assembly of filamentous bacteriophages. Prog Biophys Mol Biol. (2014) 114:80–122. 10.1016/j.pbiomolbio.2014.02.003 24582831

[72] Hay ID Lithgow T. Filamentous phages: masters of a microbial sharing economy. EMBO Rep. (2019) 20:1–24. 10.15252/embr.201847427 30952693 PMC6549030

[73] Day LA Marzec CJ Reisberg SA Casadevall A. DNA packing in filamentous bacteriophages. Annu Rev Biophys Biophys Chem. (1988) 17:509–39. 10.1146/annurev.bb.17.060188.002453 3293598

[74] Liu DJ Day LA. Pf1 virus structure: helical coat protein and DNA with paraxial phosphates. Science. (1994) 265:671–4. 10.1126/science.8036516 8036516

[75] Janmey PA Slochower DR Wang YH Wen Q Cebers A. Polyelectrolyte properties of filamentous biopolymers and their consequences in biological fluids. Soft Matter. (2014) 10:1439–49. 10.1039/c3sm50854d 24651463 PMC4009494

[76] Dogic Z Fraden S Ordered phases of filamentous viruses. Curr Opin Colloid Interface Sci. (2006) 11:47–55. 10.1016/j.cocis.2005.10.004

[77] Dogic Z. Filamentous phages as a model system in soft matter physics. Front Microbiol. (2016) 7:1013. 10.3389/fmicb.2016.01013 27446051 PMC4927585

[78] Zweckstetter M Bax A. Characterization of molecular alignment in aqueous suspensions of Pf1 bacteriophage. J Biomol NMR. (2001) 20:365–77. 10.1023/A:1011263920003 11563559

[79] Tomar S Green MM Day LA. DNA-protein interactions as the source of large-length-scale chirality evident in the liquid crystal behavior of filamentous bacteriophages. J Am Chem Soc. (2007) 129:3367–75. 10.1021/ja068498d 17316002

[80] Knezevic P Voet M Lavigne R. Prevalence of Pf1-like (pro)phage genetic elements among *Pseudomonas aeruginosa* isolates. Virology. (2015) 483:64–71. 10.1016/j.virol.2015.04.008 25965796

[81] Cumby N Davidson AR Maxwell KL. The moron comes of age. Bacteriophage. (2012) 2:225–8. 10.4161/bact.23146 23739268 PMC3594210

[82] Tsao YF Taylor VL Kala S Bondy-Denomy J Khan AN Bona D. Phage morons play an important role in *Pseudomonas aeruginosa* phenotypes. J Bacteriol. (2018) 200:e00189-18. 10.1128/JB.00189-18 30150232 PMC6199475

[83] Castang S Dove SL. Basis for the essentiality of H-NS family members in *Pseudomonas aeruginosa*. J Bacteriol. (2012) 194:5101–9. 10.1128/JB.00932-12 22821971 PMC3430348

[84] Li Y Liu X Tang K Wang P Zeng Z Guo Y. Excisionase in Pf filamentous prophage controls lysis-lysogeny decision-making in *Pseudomonas aeruginosa*. Mol Microbiol. (2019) 111:495–513. 10.1111/mmi.14170 30475408 PMC7379572

[85] Lee Y Song S Sheng L Zhu L Kim JS Wood TK. Substrate binding protein DppA1 of ABC transporter DppBCDF increases biofilm formation in *Pseudomonas aeruginosa* by inhibiting Pf5 prophage lysis. Front Microbiol. (2018) 9:30. 10.3389/fmicb.2018.00030 29416528 PMC5787571

[86] Wei Q Minh PN Dotsch A Hildebrand F Panmanee W Elfarash A. Global regulation of gene expression by OxyR in an important human opportunistic pathogen. Nucleic Acids Res. (2012) 40:4320–33. 10.1093/nar/gks017 22275523 PMC3378865

[87] Martinez E Campos-Gomez J. Pf Filamentous phage requires UvrD for replication in *Pseudomonas aeruginosa*. mSphere. (2016) 1:e00104-15. 10.1128/mSphere.00104-15 27303696 PMC4863604

[88] Chen M Samuelson JC Jiang F Muller M Kuhn A Dalbey RE. Direct interaction of YidC with the Sec-independent Pf3 coat protein during its membrane protein insertion. J Biol Chem. (2002) 277:7670–5. 10.1074/jbc.M110644200 11751917

[89] McElroy KE Hui JG Woo JK Luk AW Webb JS Kjelleberg S. Strain-specific parallel evolution drives short-term diversification during *Pseudomonas aeruginosa* biofilm formation. Proc Natl Acad Sci USA. (2014) 111:E1419–27. 10.1073/pnas.1314340111 24706926 PMC3986123

[90] Castang S McManus HR Turner KH Dove SL. H-NS family members function coordinately in an opportunistic pathogen. Proc Natl Acad Sci USA. (2008) 105:18947–52. 10.1073/pnas.0808215105 19028873 PMC2596223

[91] Winsor GL Griffiths EJ Lo R Dhillon BK Shay JA Brinkman FS. Enhanced annotations and features for comparing thousands of Pseudomonas genomes in the Pseudomonas genome database. Nucleic Acids Res. (2016) 44:D646–53. 10.1093/nar/gkv1227 26578582 PMC4702867

[92] Burgener EB Sweere JM Bach MS Secor PR Haddock N Jennings LK. Filamentous bacteriophages are associated with chronic Pseudomonas lung infections and antibiotic resistance in cystic fibrosis. Sci Transl Med. (2019) 11:eaau9748. 10.1126/scitranslmed.aau9748 30996083 PMC7021451

[93] Katoh K Kuma K Miyata T Toh H. Improvement in the accuracy of multiple sequence alignment program MAFFT. Genome Inform. (2005) 16:22–33. 10.1093/nar/gki198 16362903

[94] Price MN Dehal PS Arkin AP. FastTree 2–approximately maximum-likelihood trees for large alignments. PLoS ONE. (2010) 5:e9490. 10.1371/journal.pone.0009490 20224823 PMC2835736

[95] Takeya K Amako K. A rod-shaped Pseudomonas phage. Virology. (1966) 28:163–5. 10.1016/0042-6822(66)90317-5 4955194

[96] Canchaya C Fournous G Brussow H. The impact of prophages on bacterial chromosomes. Mol Microbiol. (2004) 53:9–18. 10.1111/j.1365-2958.2004.04113.x 15225299

[97] Klockgether J Munder A Neugebauer J Davenport CF Stanke F Larbig KD. Genome diversity of *Pseudomonas aeruginosa* PAO1 laboratory strains. J Bacteriol. (2010) 192:1113–21. 10.1128/JB.01515-09 20023018 PMC2812968

[98] Mauch RM Jensen PO Moser C Levy CE Hoiby N. Mechanisms of humoral immune response against *Pseudomonas aeruginosa* biofilm infection in cystic fibrosis. J Cyst Fibros. (2018) 17:143–52. 10.1016/j.jcf.2017.08.012 29033275

[99] Rice SA Tan CH Mikkelsen PJ Kung V Woo J Tay M. The biofilm life cycle and virulence of *Pseudomonas aeruginosa* are dependent on a filamentous prophage. ISME J. (2009) 3:271–82. 10.1038/ismej.2008.109 19005496 PMC2648530

[100] Webb JS Thompson LS James S Charlton T Tolker-Nielsen T Koch B. Cell death in *Pseudomonas aeruginosa* biofilm development. J Bacteriol. (2003) 185:4585–92. 10.1128/JB.185.15.4585-4592.2003 12867469 PMC165772

[101] Secor PR Sweere JM Michaels LA Malkovskiy AV Lazzareschi D Katznelson E. Filamentous bacteriophage promote biofilm assembly and function. Cell Host Microbe. (2015) 18:549–59. 10.1016/j.chom.2015.10.013 26567508 PMC4653043

[102] Mooij MJ Drenkard E Llamas MA Vandenbroucke-Grauls CM Savelkoul PH Ausubel FM. Characterization of the integrated filamentous phage Pf5 and its involvement in small-colony formation. Microbiology. (2007) 153(Pt 6):1790–8. 10.1099/mic.0.2006/003533-0 17526836 PMC3820363

[103] Folsom JP Richards L Pitts B Roe F Ehrlich GD Parker A. Physiology of *Pseudomonas aeruginosa* in biofilms as revealed by transcriptome analysis. BMC Microbiol. (2010) 10:294. 10.1186/1471-2180-10-294 21083928 PMC2998477

[104] Whiteley M Bangera MG Bumgarner RE Parsek MR Teitzel GM Lory S. Gene expression in *Pseudomonas aeruginosa* biofilms. Nature. (2001) 413:860–4. 10.1038/35101627 11677611

[105] Yeung AT Torfs EC Jamshidi F Bains M Wiegand I Hancock RE. Swarming of *Pseudomonas aeruginosa* is controlled by a broad spectrum of transcriptional regulators, including MetR. J Bacteriol. (2009) 191:5592–602. 10.1128/JB.00157-09 19592586 PMC2737960

[106] Platt MD Schurr MJ Sauer K Vazquez G Kukavica-Ibrulj I Potvin E. Proteomic, microarray, and signature-tagged mutagenesis analyses of anaerobic *Pseudomonas aeruginosa* at pH 6.5, likely representing chronic, late-stage cystic fibrosis airway conditions. J Bacteriol. (2008) 190:2739–58. 10.1128/JB.01683-07 18203836 PMC2293228

[107] Stoodley P Sauer K Davies DG Costerton JW. Biofilms as complex differentiated communities. Annu Rev Microbiol. (2002) 56:187–209. 10.1146/annurev.micro.56.012302.160705 12142477

[108] Whitchurch CB Tolker-Nielsen T Ragas PC Mattick JS. Extracellular DNA required for bacterial biofilm formation. Science. (2002) 295:1487. 10.1126/science.295.5559.1487 11859186

[109] Rice AR Hamilton MA Camper AK. Movement, replication, and emigration rates of individual bacteria in a biofilm. Microb Ecol. (2003) 45:163–72. 10.1007/s00248-002-1028-x 12491023

[110] Webb JS Lau M Kjelleberg S. Bacteriophage and phenotypic variation in *Pseudomonas aeruginosa* biofilm development. J Bacteriol. (2004) 186:8066–73. 10.1128/JB.186.23.8066-8073.2004 15547279 PMC529096

[111] Petrova OE Schurr JR Schurr MJ Sauer K. The novel *Pseudomonas aeruginosa* two-component regulator BfmR controls bacteriophage-mediated lysis and DNA release during biofilm development through PhdA. Mol Microbiol. (2011) 81:767–83. 10.1111/j.1365-2958.2011.07733.x 21696457 PMC3214647

[112] Secor PR Michaels LA Smigiel KS Rohani MG Jennings LK Hisert KB. Filamentous bacteriophage produced by *Pseudomonas aeruginosa* alters the inflammatory response and promotes noninvasive infection *in vivo*. Infect Immun. (2017) 85:IAI.00648-16. 10.1128/IAI.00648-16 27795361 PMC5203648

[113] Bille E Meyer J Jamet A Euphrasie D Barnier JP Brissac T. A virulence-associated filamentous bacteriophage of Neisseria meningitidis increases host-cell colonisation. PLoS Pathog. (2017) 13:e1006495. 10.1371/journal.ppat.1006495 28704569 PMC5526601

[114] Secor PR Jennings LK Michaels LA Sweere JM Singh PK Parks WC. Biofilm assembly becomes crystal clear - filamentous bacteriophage organize the *Pseudomonas aeruginosa* biofilm matrix into a liquid crystal. Microb Cell. (2015) 3:49–52. 10.15698/mic2016.01.475 28357315 PMC5354590

[115] El Haddad L Harb CP Gebara MA Stibich MA Chemaly RF. A systematic and critical review of bacteriophage therapy against multidrug-resistant ESKAPE organisms in humans. Clin Infect Dis. (2019) 69:167–78. 10.1093/cid/ciy947 30395179

[116] Gordillo Altamirano FL Barr JJ. Phage therapy in the postantibiotic era. Clin Microbiol Rev. (2019) 32:e00066-18. 10.1128/CMR.00066-18 30651225 PMC6431132

[117] Manrique P Bolduc B Walk ST van der Oost J de Vos WM Young MJ. Healthy human gut phageome. Proc Natl Acad Sci USA. (2016) 113:10400–5. 10.1073/pnas.1601060113 27573828 PMC5027468

[118] Bollyky PL Secor PR. The Innate Sense of Bacteriophages. Cell Host Microbe. (2019) 25:177–9. 10.1016/j.chom.2019.01.020 30763530

[119] Gogokhia L Buhrke K Bell R Hoffman B Brown DG Hanke-Gogokhia C. Expansion of bacteriophages is linked to aggravated intestinal inflammation and colitis. Cell Host Microbe. (2019) 25:285–99.e288. 10.1016/j.chom.2019.01.008 30763538 PMC6885004

[120] Hraiech S Bregeon F Rolain JM. Bacteriophage-based therapy in cystic fibrosis-associated *Pseudomonas aeruginosa* infections: rationale and current status. Drug Des Devel Ther. (2015) 9:3653–63. 10.2147/DDDT.S53123 26213462 PMC4509528

[121] Waters EM Neill DR Kaman B Sahota JS Clokie MRJ Winstanley C. Phage therapy is highly effective against chronic lung infections with *Pseudomonas aeruginosa*. Thorax. (2017) 72:666–7. 10.1136/thoraxjnl-2016-209265 28265031 PMC5520275

[122] Hashimoto S Pittet JF Hong K Folkesson H Bagby G Kobzik L. Depletion of alveolar macrophages decreases neutrophil chemotaxis to Pseudomonas airspace infections. Am J Physiol. (1996) 270(5 Pt 1):L819–28. 10.1152/ajplung.1996.270.5.L819 8967517

[123] Allen L Dockrell DH Pattery T Lee DG Cornelis P Hellewell PG. Pyocyanin production by *Pseudomonas aeruginosa* induces neutrophil apoptosis and impairs neutrophil-mediated host defenses *in vivo*. J Immunol. (2005) 174:3643–9. 10.4049/jimmunol.174.6.3643 15749902

[124] Alhede M Bjarnsholt T Jensen PO Phipps RK Moser C Christophersen L. *Pseudomonas aeruginosa* recognizes and responds aggressively to the presence of polymorphonuclear leukocytes. Microbiology. (2009) 155(Pt 11):3500–8. 10.1099/mic.0.031443-0 19643762

[125] Koh AY Priebe GP Ray C Van Rooijen N Pier GB. Inescapable need for neutrophils as mediators of cellular innate immunity to acute *Pseudomonas aeruginosa* pneumonia. Infect Immun. (2009) 77:5300–10. 10.1128/IAI.00501-09 19805527 PMC2786465

[126] Raoust E Balloy V Garcia-Verdugo I Touqui L Ramphal R Chignard M. *Pseudomonas aeruginosa* LPS or flagellin are sufficient to activate TLR-dependent signaling in murine alveolar macrophages and airway epithelial cells. PLoS ONE. (2009) 4:e7259. 10.1371/journal.pone.0007259 19806220 PMC2752798

[127] Maldonado RF Sa-Correia I Valvano MA. Lipopolysaccharide modification in Gram-negative bacteria during chronic infection. FEMS Microbiol Rev. (2016) 40:480–93. 10.1093/femsre/fuw007 27075488 PMC4931227

[128] Faure E Kwong K Nguyen D. *Pseudomonas aeruginosa* in chronic lung infections: how to adapt within the host? Front Immunol. (2018) 9:2416. 10.3389/fimmu.2018.02416 30405616 PMC6204374

[129] Smith EE Buckley DG Wu Z Saenphimmachak C Hoffman LR D'Argenio DA. Genetic adaptation by *Pseudomonas aeruginosa* to the airways of cystic fibrosis patients. Proc Natl Acad Sci USA. (2006) 103:8487–92. 10.1073/pnas.0602138103 16687478 PMC1482519

[130] Benmohamed F Medina M Wu YZ Maschalidi S Jouvion G Guillemot L. Toll-like receptor 9 deficiency protects mice against *Pseudomonas aeruginosa* lung infection. PLoS ONE. (2014) 9:e90466. 10.1371/journal.pone.0090466 24595157 PMC3942450

[131] Andrews T Sullivan KE. Infections in patients with inherited defects in phagocytic function. Clin Microbiol Rev. (2003) 16:597–621. 10.1128/CMR.16.4.597-621.2003 14557288 PMC207096

[132] Kurahashi K Sawa T Ota M Kajikawa O Hong K Martin TR. Depletion of phagocytes in the reticuloendothelial system causes increased inflammation and mortality in rabbits with *Pseudomonas aeruginosa* pneumonia. Am J Physiol Lung Cell Mol Physiol. (2009) 296:L198–209. 10.1152/ajplung.90472.2008 19028978 PMC2643994

[133] Jensen PO Givskov M Bjarnsholt T Moser C. The immune system vs. *Pseudomonas aeruginosa* biofilms. FEMS Immunol Med Microbiol. (2010) 59:292–305. 10.1111/j.1574-695X.2010.00706.x 20579098

[134] Kunert A Losse J Gruszin C Huhn M Kaendler K Mikkat S. Immune evasion of the human pathogen *Pseudomonas aeruginosa*: elongation factor Tuf is a factor H and plasminogen binding protein. J Immunol. (2007) 179:2979–88. 10.4049/jimmunol.179.5.2979 17709513

[135] Amiel E Lovewell RR O'Toole GA Hogan DA Berwin B. *Pseudomonas aeruginosa* evasion of phagocytosis is mediated by loss of swimming motility and is independent of flagellum expression. Infect Immun. (2010) 78:2937–45. 10.1128/IAI.00144-10 20457788 PMC2897393

[136] Lovewell RR Patankar YR Berwin B. Mechanisms of phagocytosis and host clearance of *Pseudomonas aeruginosa*. Am J Physiol Lung Cell Mol Physiol. (2014) 306:L591–603. 10.1152/ajplung.00335.2013 24464809 PMC4116407

[137] Sweere JM Van Belleghem JD Ishak H Bach MS Popescu M Sunkari V. Bacteriophage trigger antiviral immunity and prevent clearance of bacterial infection. Science. (2019) 363:eaat9691. 10.1126/science.aat9691 30923196 PMC6656896

[138] Malone JG. Role of small colony variants in persistence of *Pseudomonas aeruginosa* infections in cystic fibrosis lungs. Infect Drug Resist. (2015) 8:237–47. 10.2147/IDR.S68214 26251621 PMC4524453

[139] Kondo Y Imai M. Effect of glutaraldehyde on renal tubular function. II Selective inhibition of Cl- transport in the hamster thin ascending limb of Henle's loop. Pflugers Arch. (1987) 408:484–90. 10.1007/BF00585073 3110737

[140] Pier GB Coleman F Grout M Franklin M Ohman DE. Role of alginate O acetylation in resistance of mucoid *Pseudomonas aeruginosa* to opsonic phagocytosis. Infect Immun. (2001) 69:1895–901. 10.1128/IAI.69.3.1895-1901.2001 11179370 PMC98099

[141] Mulcahy LR Isabella VM Lewis K. *Pseudomonas aeruginosa* biofilms in disease. Microb Ecol. (2014) 68:1–12. 10.1007/s00248-013-0297-x 24096885 PMC3977026

[142] Secor PR Michaels LA Ratjen A Jennings LK Singh PK. Entropically driven aggregation of bacteria by host polymers promotes antibiotic tolerance in *Pseudomonas aeruginosa*. Proc Natl Acad Sci USA. (2018) 115:10780–5. 10.1073/pnas.1806005115 30275316 PMC6196481

[143] Collins JM Monty KJ. Cysteine biosynthesis in Salmonella typhimurium: the presence of ATP-sulfurylase and APS-kinase in various cysteine-requiring mutants. Can J Biochem. (1975) 53:1118–21. 10.1139/o75-153 173451

[144] Lee JH Del Sorbo L Khine AA de Azavedo J Low DE Bell D. Modulation of bacterial growth by tumor necrosis factor-alpha *in vitro* and *in vivo*. Am J Respir Crit Care Med. (2003) 168:1462–70. 10.1164/rccm.200302-303OC 12958055

[145] Aronow R Danon D Shahar A Aronson M. Electron microscopy of *in vitro* endocytosis of T2 phage by cells from rabbit peritoneal exudate. J Exp Med. (1964) 120:943–54. 10.1084/jem.120.5.943 14247730 PMC2137874

[146] Wenger SL Turner JH Petricciani JC. The cytogenetic, proliferative and viability effects of four bacteriophages on human lymphocytes. In Vitro. (1978) 14:543–9. 10.1007/BF02616097 680773

[147] Nguyen S Baker K Padman BS Patwa R Dunstan RA Weston TA. Bacteriophage Transcytosis Provides a Mechanism To Cross Epithelial Cell Layers. MBio. (2017) 8:e01874-17. 10.1128/mBio.01874-17 29162715 PMC5698557

[148] Shapouri-Moghaddam A Mohammadian S Vazini H Taghadosi M Esmaeili SA Mardani F. Macrophage plasticity, polarization, and function in health and disease. J Cell Physiol. (2018) 233:6425–40. 10.1002/jcp.26429 29319160 PMC13482560

[149] Gorski A Miedzybrodzki R Borysowski J Dabrowska K Wierzbicki P Ohams M. Phage as a modulator of immune responses: practical implications for phage therapy. Adv Virus Res. (2012) 83:41–71. 10.1016/B978-0-12-394438-2.00002-5 22748808

[150] Krut O Bekeredjian-Ding I. Contribution of the Immune Response to Phage Therapy. J Immunol. (2018) 200:3037–44. 10.4049/jimmunol.1701745 29685950

[151] Van Belleghem JD Dabrowska K Vaneechoutte M Barr JJ and Bollyky PL. Interactions between Bacteriophage, Bacteria, and the Mammalian Immune System. Viruses. (2018) 11:10. 10.3390/v11010010 30585199 PMC6356784

[152] Tiwari BR Kim S Rahman M Kim J. Antibacterial efficacy of lytic Pseudomonas bacteriophage in normal and neutropenic mice models. J Microbiol. (2011) 49:994–9. 10.1007/s12275-011-1512-4 22203564

[153] Pincus NB Reckhow JD Saleem D Jammeh ML Datta SK Myles IA. Strain specific phage Treatment for *Staphylococcus aureus* infection is influenced by host immunity and site of infection. PLoS ONE. (2015) 10:e0124280. 10.1371/journal.pone.0124280 25909449 PMC4409319

[154] Leung CYJ Weitz JS. Modeling the synergistic elimination of bacteria by phage and the innate immune system. J Theor Biol. (2017) 429:241–52. 10.1016/j.jtbi.2017.06.037 28668337

[155] Roach DR Leung CY Henry M Morello E Singh D Di Santo JP. Synergy between the host immune system and bacteriophage is essential for successful phage therapy against an acute respiratory pathogen. Cell Host Microbe. (2017) 22:38–47.e34. 10.1016/j.chom.2017.06.018 28704651

[156] Letrado P Corsini B Diez-Martinez R Bustamante N Yuste JE Garcia P. Bactericidal synergism between antibiotics and phage endolysin Cpl-711 to kill multidrug-resistant pneumococcus. Future Microbiol. (2018) 13:1215–23. 10.2217/fmb-2018-0077 30238774 PMC6190277

[157] Kaur S Harjai K Chhibber S. Bacteriophage-aided intracellular killing of engulfed methicillin-resistant *Staphylococcus aureus* (MRSA) by murine macrophages. Appl Microbiol Biotechnol. (2014) 98:4653–61. 10.1007/s00253-014-5643-5 24633444

[158] Jonczyk-Matysiak E Lusiak-Szelachowska M Klak M Bubak B Miedzybrodzki R Weber-Dabrowska B. The effect of bacteriophage preparations on intracellular killing of bacteria by phagocytes. J Immunol Res. (2015) 2015:482863. 10.1155/2015/482863 26783541 PMC4689956

[159] Lehti TA Pajunen MI Skog MS Finne J. Internalization of a polysialic acid-binding *Escherichia coli* bacteriophage into eukaryotic neuroblastoma cells. Nat Commun. (2017) 8:1915. 10.1038/s41467-017-02057-3 29203765 PMC5715158

[160] Weber-Dabrowska B Zimecki M Mulczyk M. Effective phage therapy is associated with normalization of cytokine production by blood cell cultures. Arch Immunol Ther Exp. (Warsz). (2000) 48:31–7. 10722229

[161] Van Belleghem JD Clement F Merabishvili M Lavigne R Vaneechoutte M. Pro- and anti-inflammatory responses of peripheral blood mononuclear cells induced by *Staphylococcus aureus* and *Pseudomonas aeruginosa* phages. Sci Rep. (2017) 7:8004. 10.1038/s41598-017-08336-9 28808331 PMC5556114

[162] Zhang L Hou X Sun L He T Wei R Pang M. *Staphylococcus aureus* bacteriophage suppresses LPS-induced inflammation in MAC-T bovine mammary epithelial cells. Front Microbiol. (2018) 9:1614. 10.3389/fmicb.2018.01614 30083140 PMC6064726

[163] Miernikiewicz P Dabrowska K Piotrowicz A Owczarek B Wojas-Turek J Kicielinska J. T4 phage and its head surface proteins do not stimulate inflammatory mediator production. PLoS ONE. (2013) 8:e71036. 10.1371/journal.pone.0071036 23976975 PMC3745418

[164] Park K Cha KE Myung H. Observation of inflammatory responses in mice orally fed with bacteriophage T7. J Appl Microbiol. (2014) 117:627–33. 10.1111/jam.12565 24916438

[165] Miedzybrodzki R Switala-Jelen K Fortuna W Weber-Dabrowska B Przerwa A Lusiak-Szelachowska M. Bacteriophage preparation inhibition of reactive oxygen species generation by endotoxin-stimulated polymorphonuclear leukocytes. Virus Res. (2008) 131:233–42. 10.1016/j.virusres.2007.09.013 17996972

[166] Miernikiewicz P Klopot A Soluch R Szkuta P Keska W Hodyra-Stefaniak K. T4 phage tail adhesin Gp12 counteracts LPS-induced inflammation *in vivo*. Front Microbiol. (2016) 7:1112. 10.3389/fmicb.2016.01112 27471503 PMC4943950

[167] Foxman EF Iwasaki A. Genome-virome interactions: examining the role of common viral infections in complex disease. Nat Rev Microbiol. (2011) 9:254–64. 10.1038/nrmicro2541 21407242 PMC3678363

[168] Duerkop BA Hooper LV. Resident viruses and their interactions with the immune system. Nat Immunol. (2013) 14:654–9. 10.1038/ni.2614 23778792 PMC3760236

[169] Matsui H Randell SH Peretti SW Davis CW Boucher RC. Coordinated clearance of periciliary liquid and mucus from airway surfaces. J Clin Invest. (1998) 102:1125–31. 10.1172/JCI2687 9739046 PMC509095

[170] Kreda SM Mall M Mengos A Rochelle L Yankaskas J Riordan JR. Characterization of wild-type and deltaF508 cystic fibrosis transmembrane regulator in human respiratory epithelia. Mol Biol Cell. (2005) 16:2154–67. 10.1091/mbc.e04-11-1010 15716351 PMC1087225

[171] Tarran R Button B Boucher RC. Regulation of normal and cystic fibrosis airway surface liquid volume by phasic shear stress. Ann Rev Physiol. (2006) 68:543–61. 10.1146/annurev.physiol.68.072304.112754 16460283

[172] Stoltz DA Meyerholz DK Pezzulo AA Ramachandran S Rogan MP Davis GJ. Cystic fibrosis pigs develop lung disease and exhibit defective bacterial eradication at birth. Sci Transl Med. (2010) 2:29ra31. 10.1126/scitranslmed.3000928 20427821 PMC2889616

[173] Hartl D Gaggar A Bruscia E Hector A Marcos V Jung A. Innate immunity in cystic fibrosis lung disease. J Cyst Fibros. (2012) 11:363–82. 10.1016/j.jcf.2012.07.003 22917571

[174] Hoegger MJ Fischer AJ McMenimen JD Ostedgaard LS Tucker AJ Awadalla MA. Impaired mucus detachment disrupts mucociliary transport in a piglet model of cystic fibrosis. Science. (2014) 345:818–22. 10.1126/science.1255825 25124441 PMC4346163

[175] FitzSimmons SC. The changing epidemiology of cystic fibrosis. J Pediatr. (1993) 122:1–9. 10.1016/S0022-3476(05)83478-X 8419592

[176] Johansen HK Høiby N. Seasonal onset of initial colonisation and chronic infection with *Pseudomonas aeruginosa* in patients with cystic fibrosis in Denmark. Thorax. (1992) 47:109–11. 10.1136/thx.47.2.109 1549817 PMC463585

[177] Burns JL Gibson RL McNamara S Yim D Emerson J Rosenfeld M. Longitudinal assessment of *Pseudomonas aeruginosa* in young children with cystic fibrosis. J Infect Dis. (2001) 183:444–52. 10.1086/318075 11133376

[178] Cystic Fibrosis Foundation (2016). Cystic Fibrosis Foundation Patient Registry 2015 Annual Data Report.

[179] UK Cystic Fibrosis Registry (2017). UK Cystic Fibrosis Registry Annual Data Report 2016.

[180] Nixon GM Armstrong DS Carzino R Carlin JB Olinsky A Robertson CF. Clinical outcome after early *Pseudomonas aeruginosa* infection in cystic fibrosis. J Pediatr. (2001) 138:699–704. 10.1067/mpd.2001.112897 11343046

[181] Rosenfeld M Gibson RL McNamara S Emerson J Burns JL Castile R. Early pulmonary infection, inflammation, and clinical outcomes in infants with cystic fibrosis. Pediatr Pulmonol. (2001) 32:356–66. 10.1002/ppul.1144 11596160

[182] Konstan MW Morgan WJ Butler SM Pasta DJ Craib ML Silva SJ. Risk factors for rate of decline in forced expiratory volume in one second in children and adolescents with cystic fibrosis. J Pediatr. (2007) 151:134–9, 139.e131. 10.1016/j.jpeds.2007.03.006 17643762

[183] Emerson J McNamara S Buccat AM Worrell K Burns JL. Changes in cystic fibrosis sputum microbiology in the United States between 1995 and 2008. Pediatr Pulmonol. (2010) 45:363–70. 10.1002/ppul.21198 20232473

[184] Doring G Flume P Heijerman H Elborn JS Consensus Study G. Treatment of lung infection in patients with cystic fibrosis: current and future strategies. J Cyst Fibros. (2012) 11:461–79. 10.1016/j.jcf.2012.10.004 23137712

[185] Mogayzel PJ Jr Naureckas ET Robinson KA Brady C Guill M Lahiri T. Cystic Fibrosis Foundation pulmonary guideline. pharmacologic approaches to prevention and eradication of initial Pseudomonas aeruginosa infection. Ann Am Thorac Soc. (2014) 11:1640–50. 10.1513/AnnalsATS.201404-166OC 25549030

[186] Burgener EB Yacob AA Bollyky P Milla CE 99 Pf bacteriophage (Pf) in *Pseudomonas aeruginosa* (Pa) biofilms is associated with increased elastase in the sputum of patients with cystic fibrosis (CF). J Cystic Fibrosis. (2017) 16:S90 10.1016/S1569-1993(17)30463-0

[187] Chang MS McNinch J Basu R Simonet S. Cloning and characterization of the human neutrophil-activating peptide (ENA-78) gene. J Biol Chem. (1994) 269:25277–82. 7929219

[188] Koch AE Kunkel SL Harlow LA Mazarakis DD Haines GK Burdick MD. Epithelial neutrophil activating peptide-78: a novel chemotactic cytokine for neutrophils in arthritis. J Clin Invest. (1994) 94:1012–8. 10.1172/JCI117414 8083342 PMC295150

[189] Bokarewa M Nagaev I Dahlberg L Smith U Tarkowski A. Resistin, an adipokine with potent proinflammatory properties. J Immunol. (2005) 174:5789–95. 10.4049/jimmunol.174.9.5789 15843582

[190] Cooper AM Khader SA. IL-12p40: an inherently agonistic cytokine. Trends Immunol. (2007) 28:33–8. 10.1016/j.it.2006.11.002 17126601

[191] Schaefer U Voloshanenko O Willen D Walczak H. TRAIL: a multifunctional cytokine. Front Biosci. (2007) 12:3813–24. 10.2741/2354 17485341

[192] Dandachi N Kelly NJ Wood JP Burton CL Radder JE Leme AS. Macrophage elastase induces TRAIL-mediated tumor cell death through its carboxy-terminal domain. Am J Respir Crit Care Med. (2017) 196:353–63. 10.1164/rccm.201606-1150OC 28345958 PMC5549863

[193] Priebe GP Goldberg JB Vaccines for *Pseudomonas aeruginosa*: A long and winding road. Expert Rev Vaccines. (2014) 3:507–19. 10.1586/14760584.2014.890053 PMC4521563 24575895

[194] Jerne NK. Bacteriophage inactivation by antiphage serum diluted in distilled water. Nature. (1952) 169:117–8. 10.1038/169117b0 14910703

[195] Jerne NK. The presence in normal serum of specific antibody against bacteriophage T4 and its increase during the earliest stages of immunization. J Immunol. (1956) 76:209–16. 13306956

[196] Zaczek M Lusiak-Szelachowska M Jonczyk-Matysiak E Weber-Dabrowska B Miedzybrodzki R Owczarek B. Antibody production in response to staphylococcal MS-1 phage cocktail in patients undergoing phage therapy. Front Microbiol. (2016) 7:1681. 10.3389/fmicb.2016.01681 27822205 PMC5075762

[197] Dabrowska K Miernikiewicz P Piotrowicz A Hodyra K Owczarek B Lecion D. Immunogenicity studies of proteins forming the T4 phage head surface. J Virol. (2014) 88:12551–7. 10.1128/JVI.02043-14 25142581 PMC4248953

[198] Bruttin A Brussow H. Human volunteers receiving *Escherichia coli* phage T4 orally: a safety test of phage therapy. Antimicrob Agents Chemother. (2005) 49:2874–8. 10.1128/AAC.49.7.2874-2878.2005 15980363 PMC1168693

[199] Levy SB Marshall B. Antibacterial resistance worldwide: causes, challenges and responses. Nat Med. (2004) 10:S122–9. 10.1038/nm1145 15577930

[200] Summers WC. Bacteriophage therapy. Annu Rev Microbiol. (2001) 55:437–51. 10.1146/annurev.micro.55.1.437 11544363

[201] Bertoye A Gaillard L Courtieu AL. [Adapted bacteriophages in the treatment of infections caused by antibiotic-resistant microorganisms]. J Med Lyon. (1959) 40:465–71. 13655000

[202] Soothill J. Use of bacteriophages in the treatment of *Pseudomonas aeruginosa* infections. Expert Rev Anti Infect Ther. (2013) 11:909–15. 10.1586/14787210.2013.826990 24053272

[203] Chang RY Wong J Mathai A Morales S Kutter E Britton W. Production of highly stable spray dried phage formulations for treatment of *Pseudomonas aeruginosa* lung infection. Eur J Pharm Biopharm. (2017) 121:1–13. 10.1016/j.ejpb.2017.09.002 28890220 PMC5650506

[204] Loc-Carrillo C Abedon ST. Pros and cons of phage therapy. Bacteriophage. (2011) 1:111–4. 10.4161/bact.1.2.14590 22334867 PMC3278648

[205] Shiley JR Comfort KK Robinson JB. Immunogenicity and antimicrobial effectiveness of *Pseudomonas aeruginosa* specific bacteriophage in a human lung *in vitro* model. Appl Microbiol Biotechnol. (2017) 101:7977–85. 10.1007/s00253-017-8504-1 28914348

[206] Cadwell K. The virome in host health and disease. Immunity. (2015) 42:805–13. 10.1016/j.immuni.2015.05.003 25992857 PMC4578625

[207] Pride DT Salzman J Haynes M Rohwer F Davis-Long C White RA III. Evidence of a robust resident bacteriophage population revealed through analysis of the human salivary virome. ISME J. (2012) 6:915–26. 10.1038/ismej.2011.169 22158393 PMC3329113

[208] Yang J Yang F Ren L Xiong Z Wu Z Dong J. Unbiased parallel detection of viral pathogens in clinical samples by use of a metagenomic approach. J Clin Microbiol. (2011) 49:3463–9. 10.1128/JCM.00273-11 21813714 PMC3187305

[209] Carding SR Davis N Hoyles L. Review article: the human intestinal virome in health and disease. Aliment Pharmacol Ther. (2017) 46:800–15. 10.1111/apt.14280 28869283 PMC5656937

[210] Enault F Briet A Bouteille L Roux S Sullivan MB Petit MA. Phages rarely encode antibiotic resistance genes: a cautionary tale for virome analyses. ISME J. (2017) 11:237–47. 10.1038/ismej.2016.90 27326545 PMC5315482

[211] Willner D Furlan M Haynes M Schmieder R Angly FE Silva J. Metagenomic analysis of respiratory tract DNA viral communities in cystic fibrosis and non-cystic fibrosis individuals. PLoS ONE. (2009) 4:e7370. 10.1371/journal.pone.0007370 19816605 PMC2756586

[212] Dickson RP Huffnagle GB. The lung microbiome: new principles for respiratory bacteriology in health and disease. PLoS Pathog. (2015) 11:e1004923. 10.1371/journal.ppat.1004923 26158874 PMC4497592

[213] Jorth P Staudinger BJ Wu X Hisert KB Hayden H Garudathri J. Regional isolation drives bacterial diversification within cystic fibrosis lungs. Cell Host Microbe. (2015) 18:307–19. 10.1016/j.chom.2015.07.006 26299432 PMC4589543

[214] Fodor AA Klem ER Gilpin DF Elborn JS Boucher RC Tunney MM. The adult cystic fibrosis airway microbiota is stable over time and infection type, and highly resilient to antibiotic treatment of exacerbations. PLoS ONE. (2012) 7:e45001. 10.1371/journal.pone.0045001 23049765 PMC3458854

[215] Huang YJ LiPuma JJ. The microbiome in cystic fibrosis. Clin Chest Med. (2016) 37:59–67. 10.1016/j.ccm.2015.10.003 26857768 PMC5154676

[216] Penner JC Ferreira JAG Secor PR Sweere JM Birukova MK Joubert LM. Pf4 bacteriophage produced by *Pseudomonas aeruginosa* inhibits *Aspergillus fumigatus* metabolism via iron sequestration. Microbiology. (2016) 162:1583–94. 10.1099/mic.0.000344 27473221

[217] Nazik H Joubert LM Secor PR Sweere JM Bollyky PL Sass G. Pseudomonas phage inhibition of *Candida albicans*. Microbiology. (2017) 163:1568–77. 10.1099/mic.0.000539 28982395

[218] James CE Davies EV Fothergill JL Walshaw MJ Beale CM Brockhurst MA. Lytic activity by temperate phages of *Pseudomonas aeruginosa* in long-term cystic fibrosis chronic lung infections. ISME J. (2015) 9:1391–8. 10.1038/ismej.2014.223 25461970 PMC4351911

[219] Cohen D Melamed S Millman A Shulman G Oppenheimer-Shaanan Y Kacen A. Cyclic GMP-AMP signalling protects bacteria against viral infection. Nature. (2019) 574:691–5. 10.1038/s41586-019-1605-5 31533127

[220] Mougous JD Cuff ME Raunser S Shen A Zhou M Gifford CA. A virulence locus of *Pseudomonas aeruginosa* encodes a protein secretion apparatus. Science. (2006) 312:1526–30. 10.1126/science.1128393 16763151 PMC2800167

[221] Hockett KL Renner T Baltrus DA. Independent co-option of a tailed bacteriophage into a killing complex in pseudomonas. MBio. (2015) 6:e00452. 10.1128/mBio.00452-15 26265717 PMC4542187

[222] Costantini TW Putnam JG Sawada R Baird A Loomis WH Eliceiri BP. Targeting the gut barrier: identification of a homing peptide sequence for delivery into the injured intestinal epithelial cell. Surgery. (2009) 146:206–12. 10.1016/j.surg.2009.05.007 19628075 PMC4251594

[223] Ivanenkov V Felici F Menon AG. Uptake and intracellular fate of phage display vectors in mammalian cells. Biochim Biophys Acta. (1999) 1448:450–62. 10.1016/S0167-4889(98)00162-1 9990297

[224] Ochs HD Davis SD Wedgwood RJ. Immunologic responses to bacteriophage phi-X 174 in immunodeficiency diseases. J Clin Invest. (1971) 50:2559–68. 10.1172/JCI106756 5129308 PMC292205

[225] Kamme C. Antibodies against staphylococcal bacteriophages in human sera. I Assay of antibodies in healthy individuals and in patients with staphylococcal infections. Acta Pathol Microbiol Scand B Microbiol Immunol. (1973) 81:741–8. 10.1111/j.1699-0463.1973.tb02269.x 4273800

[226] Hodyra-Stefaniak K Miernikiewicz P Drapala J Drab M Jonczyk-Matysiak E Lecion D. Mammalian host-versus-phage immune response determines phage fate *in vivo*. Sci Rep. (2015) 5:14802. 10.1038/srep14802 26440922 PMC4594097

